# Oncolytic adenovirus decreases the proportion of TIM-3^+^ subset of tumor-infiltrating CD8^+^ T cells with correlation to improved survival in patients with cancer

**DOI:** 10.1136/jitc-2021-003490

**Published:** 2022-02-22

**Authors:** Ilkka Liikanen, Saru Basnet, Dafne C A Quixabeira, Kristian Taipale, Otto Hemminki, Minna Oksanen, Matti Kankainen, Juuso Juhila, Anna Kanerva, Timo Joensuu, Siri Tähtinen, Akseli Hemminki

**Affiliations:** 1 Division of Biological Sciences, Section of Molecular Biology, University of California San Diego, San Diego, California, USA; 2 Department of Oncology, Helsinki University Hospital Comprehensive Cancer Center, University of Helsinki, Helsinki, Finland; 3 Translational Immunology Research Program, Cancer Gene Therapy Group, University of Helsinki, Helsinki, Finland; 4 Cancer Gene Therapy Group, Translational Immunology Research Program, University of Helsinki, Helsinki, Finland; 5 Division of Urologic Oncology, Department of Surgical Oncology, University of Toronto, Toronto, Ontario, Canada; 6 Department of Urology, Helsinki University Hospital, University of Helsinki, Helsinki, Finland; 7 Medical and Clinical Genetics, Helsinki University Hospital, University of Helsinki, Helsinki, Finland; 8 Translational Immunology Research Program and Department of Clinical Chemistry, University of Helsinki, Helsinki, Finland; 9 Department of Obstetrics and Gynecology, Helsinki University Hospital, University of Helsinki, Helsinki, Finland; 10 Docrates Cancer Hospital, Helsinki, Finland; 11 TILT Biotherapeutics Ltd, Helsinki, Finland

**Keywords:** immunotherapy, oncolytic viruses, adaptive immunity, biomarkers, tumor, translational medical research

## Abstract

**Background:**

Oncolytic viruses are a potent form of active immunotherapy, capable of invoking antitumor T-cell responses. Meanwhile, less is known about their effects on immune checkpoints, the main targets for passive immunotherapy of cancer. T-cell immunoglobulin and mucin domain-3 (TIM-3) is a coinhibitory checkpoint driving T-cell exhaustion in cancer. Here we investigated the effects of oncolytic adenovirus on the TIM-3 checkpoint on tumor-infiltrating immune cells and clinical impact in patients with cancer receiving oncolytic immunotherapy.

**Methods:**

Modulation of TIM-3 expression on tumor-infiltrating immune cells was studied preclinically in B16 melanoma following intratumoral treatment with Ad5/3∆24-granulocyte-macrophage colony-stimulating factor oncolytic adenovirus. We conducted a retrospective longitudinal analysis of 15 patients with advanced-stage cancer with tumor-site biopsies before and after oncolytic immunotherapy, treated in the Advanced Therapy Access Program (ISRCTN10141600, April 5, 2011). Following patient stratification with regard to TIM-3 (increase vs decrease in tumors), overall survival and imaging/marker responses were evaluated by log-rank and Fisher’s test, while coinhibitory receptors/ligands, transcriptomic changes and tumor-reactive and tumor-infltrating immune cells in biopsies and blood samples were studied by microarray rank-based statistics and immunoassays.

**Results:**

Preclinically, TIM-3^+^ tumor-infiltrating lymphocytes (TILs) in B16 melanoma showed an exhausted phenotype, whereas oncolytic adenovirus treatment significantly reduced the proportion of TIM-3^+^ TIL subset through recruitment of less-exhausted CD8^+^ TIL. Decrease of TIM-3 was observed in 60% of patients, which was associated with improved overall survival over TIM-3 increase patients (p=0.004), together with evidence of clinical benefit by imaging and blood analyses. Coinhibitory T-cell receptors and ligands were consistently associated with TIM-3 changes in gene expression data, while core transcriptional exhaustion programs and T-cell dysfunction were enriched in patients with TIM-3 increase, thus identifying patients potentially benefiting from checkpoint blockade. In striking contrast, patients with TIM-3 decrease displayed an acute inflammatory signature, redistribution of tumor-reactive CD8^+^ lymphocytes and higher influx of CD8^+^ TIL into tumors, which were associated with the longest overall survival, suggesting benefit from active immunotherapy.

**Conclusions:**

Our results indicate a key role for the TIM-3 immune checkpoint in oncolytic adenoviral immunotherapy. Moreover, our results identify TIM-3 as a potential biomarker for oncolytic adenoviruses and create rationale for combination with passive immunotherapy for a subset of patients.

## Introduction

Oncolytic viruses are rapidly entering the clinical arena, with herpes simplex virus talimogene laherparepvec approved for treatment of metastatic melanoma,^1^ H101 approved for head and neck cancer,^2^ and multiple phase II and III clinical trials ongoing. Mechanism of action of oncolytic immunotherapy includes direct cancer cell death (oncolysis), spreading of tumor epitopes to mount de novo immune responses, and release of immunogenic danger signals that attract effector immune cells.^3^ Arming the virus with immunostimulatory molecules has the potential to further enhance these aspects.^4–7^ Consequently, activation of CD8^+^ antitumor immunity is attainable even against metastatic advanced tumors, as demonstrated for serotype chimeric oncolytic adenoviruses Ad5/3-hTERT-CD40L^6^ and Ad5/3∆24-granulocyte-macrophage colony stimulating factor (GMCSF) recently.^8^ Hence, oncolytic viruses are regarded as active forms of immunotherapy with the potential of turning immunoprivileged ‘cold tumors’ into inflamed ‘hot tumors’.^4^ However, less is known about the effects of oncolytic viruses on T-cell exhaustion, a key aspect of immunosuppression in inflamed tumors.

Discovered initially in chronic viral infections, T-cell exhaustion has been established as a hallmark of immune evasion exploited by malignant tumors. Chronic antigen stimulus, accumulation of coinhibitory receptors, and loss of costimulation lead to progressive CD8^+^ T-cell dysfunction.^9^ T-cell immunoglobulin and mucin domain-3 (TIM-3) is a coinhibitory receptor expressed on immune effector and myeloid cells.^10^ Tumor-site expression of TIM-3 correlates with disease progression across cancer types,^11 12^ while knockdown or silencing of *HAVCR2* (encoding and referred to as TIM-3) reinvigorates CD8^+^ T cells against advanced solid tumors,^10^ establishing its role as a key immune checkpoint of adaptive immunity.

Ligands for TIM-3 receptor include high-mobility group box-1 protein (HMGB1), which we previously characterized as a predictive serum biomarker for oncolytic immunotherapy.^13^ Moreover, transcriptomic analysis of patient tumors at baseline identified elevated TIM-3 as a potential negative prognostic factor,^14^ together suggesting that TIM-3 signaling may contribute to the immunotherapeutic activity of oncolytic adenovirus. Indeed, targeting TIM-3 has the potential to improve not only passive immunotherapy with other checkpoint inhibitors but also active immunotherapy by cancer vaccines.^15 16^ Consequently, multiple clinical trials targeting the TIM-3 checkpoint are under way, with emphasis on combination immunotherapy.^10^ Thus, it remains imperative to study in turn how active immunotherapy by oncolytic viruses impacts such immune checkpoints and T-cell exhaustion. One such potential mechanism is through recruitment of less-exhausted T-cell clones into tumors, which may also further remodel the tumor microenvironment permissive for active immunotherapy, as recently demonstrated for an oncolytic vaccinia virus preclinically.^17^

In this translational study, we show that oncolytic adenovirus treatment can reduce TIM-3 levels on CD8^+^ tumor-infiltrating lymphocyte (TIL) in both animals and in patient tumors, with correlation to improved overall survival. The effect was found to be dependent on newly recruited CD8+ TIL in mice, which alleviates the T-cell exhaustion phenotype. We further study immunological and clinical effects of TIM-3 modulation and find evidence of reciprocal impact on checkpoint signaling pathways, T-cell exhaustion, redistribution, and influx of CD8^+^ TIL subsets in patients, with association to survival benefits in TIM-3 decrease patients. Meanwhile, patients with TIM-3 increase show an exhausted dysfunctional T-cell profile with less impact on CD8^+^ immunity by oncolytic immunotherapy. These patients could potentially benefit from combination checkpoint blockade. Our results further suggest putative drivers of T-cell exhaustion phenotype in virus-treated patients and identify TIM-3 expression as a potential dynamic biomarker for oncolytic immunotherapy.

## Materials and methods

### Animal experiments

To establish tumors, 2.5×10^5^ B16-OVA cells were injected subcutaneously in the right flank of immunocompetent C57BL/6 female mice 6–8 weeks old. Seven days later, mice were either euthanized for TIL analysis or randomized for intratumoral treatment of either 1.0×10^9^ virus particles of oncolytic adenovirus Ad5/3-∆24-GMCSF or Ad5/3-∆24 or saline for 6 consecutive days, followed by investigator-blinded immune cell analysis of all tumors at 12–14 days after the onset of oncolytic immunotherapy. Indicated mice received daily intraperitoneal injections of 1 mg/kg of FTY720 (cat.#SML0700, Sigma-Aldrich) or vehicle (1.3% dimethyl sulfoxide (DMSO) in phosphate-buffered saline) starting 4 days before therapy onset. Animal experiments were approved by the University of Helsinki and the provincial government of Southern Finland.

### Tissue processing and flow cytometry

Following mechanical dissociation and red blood cell lysis, single-cell tumor suspensions were stained according to manufacturer’s instructions by commercial fluorescent dye-conjugated antibodies as detailed in the online supplemental Materials and methods and analyzed by BD Accuri C6 or LSRFortessa X-20 flow cytometer (BD Biosciences) and FlowJo software V.10.5.3 (Tree Star).

### Oncolytic adenoviruses

Oncolytic adenoviruses listed in online supplemental table S1 are genetically modified for tumor selectivity and to express immunostimulatory transgenes (GMCSF or CD40L), and their construction, preclinical and clinical use have been published.^6 8 18–20^

10.1136/jitc-2021-003490.supp1 Supplementary data

### Patient treatments and surveillance

Patient with metastatic solid tumors progressing after conventional therapies, eligible as reported,^13^ received oncolytic adenovirus (online supplemental table S1) in the Advanced Therapy Access Program (ISRCTN10141600, April 5, 2011) as described earlier,^13^ regulated by Finnish Medicines Agency FIMEA as determined by the European Committee Regulations No. 726/2004 and 1394/2007. Patients received concomitant low-dose chemotherapy (table 1) as a virus sensitizer.^21^ Surveillance by PET-CT and tumor markers, if elevated at baseline, were performed before and at 69 days’ median (±9.6 days (SEM)) after the onset of oncolytic immunotherapy, which corresponded to the post-treatment biopsy date (70 days±8.6 days (SEM)). Radiological/marker evaluation was applied to overall disease status as reported^22^ and indicated in online supplemental table S1.

**Table 1:** Patient characteristics and treatments in TIM-3 expression change groups

Clinical parameter		TIM-3 decrease (n=9)	TIM-3 increase (n=6)	Total (N=15)	Significance* (decr. vs incr.)
Baseline/treatment characteristic		Patients, n (% of total)			P value
Gender	Female	7 (77.8)	4 (66.7)	11 (73.3)	ns
	Male	2 (22.2)	2 (33.3)	4 (26.7)	ns
Age group	Adult (25–65 years)	6 (66.7)	4 (66.7)	10 (66.7)	ns
	Elderly (>65 years)	3 (33.3)	2 (33.3)	5 (33.3)	ns
WHO perf. status (0–5)	0	1 (11.1)	0 (0.0)	1 (6.7)	ns
	1	4 (44.4)	2 (33.3)	6 (40.0)	ns
	2	4 (44.4)	3 (50.0)	7 (46.7)	ns
	3	0 (0.0)	1 (16.7)	1 (6.7)	ns
Tumor type	Pancreatic	1 (11.1)	1 (16.7)	2 (13.3)	ns
	Colorectal	1 (11.1)	1 (16.7)	2 (13.3)	ns
	Prostate	1 (11.1)	0 (0.0)	1 (6.7)	ns
	Mesothelioma	0 (0.0)	1 (16.7)	1 (6.7)	ns
	Melanoma	1 (11.1)	0 (0.0)	1 (6.7)	ns
	Lung (NSCLC)	1 (11.1)	0 (0.0)	1 (6.7)	ns
	Cervical	1 (11.1)	0 (0.0)	1 (6.7)	ns
	Ovarian	2 (22.2)	2 (33.3)	4 (26.7)	ns
	Breast	1 (11.1)	1 (16.7)	2 (13.3)	ns
Sample material	Biopsy	7 (77.8)	3 (50.0)	10 (66.7)	ns
	Ascites	1 (11.1)	2 (33.3)	3 (20.0)	ns
	Pleural	1 (11.1)	1 (16.7)	2 (13.3)	ns
Oncolytic virus arming	GMCSF	6 (66.7)	4 (66.7)	10 (66.7)	ns
	CD40L	1 (11.1)	2 (33.3)	3 (20.0)	ns
	No transgene	2 (22.2)	0 (0.0)	2 (13.3)	ns
Virus sensitizer†	Cyclophosphamide	8 (88.9)	5 (83.3)	13 (86.7)	ns
	Temozolomide+CP	1 (11.1)	1 (16.7)	2 (13.3)	ns

*Patients were stratified into TIM-3 decr. and TIM-3 incr. groups based on direction of TIM-3 expression change: Fisher’s exact test was used for categorical variables between TIM-3 groups, while unpaired t-test was also tested for the linear variable age.

†Virus sensitizers include low-dose chemotherapy regimens routinely used in an adjuvant setting with oncolytic viruses: Low-dose cyclophosphamide (CP) was used for selective reduction of regulatory T cells.^21^ CP was administered either metronomically orally, starting 1 week before virus injection and continued until progression, or intravenously on the day of virus treatment, or as a combination of these. Low-dose pulse of temozolomide was administered concurrently orally (1 week before, 1–2 weeks after the virus treatment, or as a combination of these) to induce immunogenic cell death, as reported.^21^

CD40L, CD40 ligand; CP, cyclophosphamide; decr., drecease; GMCSF, granulocyte-macrophage colony-stimulating factor; incr., increase; ns, not significant; NSCLC, non-small cell lung carcinoma; TIM-3, T-cell immunoglobulin and mucin domain-3.

### Patient and public involvement statement

Patients were not involved in the conduction of research.

### Patient biopsy samples

Core needle or liquid biopsies, depending on the location of the tumor (online supplemental table S1), were taken at baseline and after a median of 70 days (average of 8 weeks) of oncolytic virus therapy, in ultrasound guidance. Tumor-affected ascites and pleural effusions were freshly pelleted and stored in RNALater. Histologically verified tumor biopsies were either stored in RNALater (Life Technologies) or fixed in formalin for immunohistochemisty.

### RNA microarrays and data analysis

RNA extraction, hybridization, and data analysis are detailed in the online supplemental materials.

### Immunohistochemistry

Immunohistochemistry on tumor sections was performed as described earlier.^14^

### Clinical PBMC analyses

Frozen PBMCs, obtained on the day of biopsies, were stained with fluorescent-dye conjugated commercial antibodies and analyzed by flow cytometry as previously described,^23^ or submitted to IFN-γ ELISPOT analysis against the ubiquitous tumor-antigen survivin, as described earlier.^21^

### Serum HMGB1 assay

ELISA for HMGB1 protein was performed as previously described.^13^

### Statistical analysis

Preclinical data were analyzed using Pearson’s coefficient and unpaired t-tests, while clinical data were additionally analyzed by log-rank and Fisher’s exact tests (GraphPad Prism V.6.0), as indicated in each figure legend. Statistical analysis of transcriptomic data is detailed in the online supplemental Materials and methods. P values of <0.05 were considered statistically significant.

## Results

### Oncolytic adenovirus treatment reduces the proportion of TIM-3 expression on TILs

**Figure 1:**
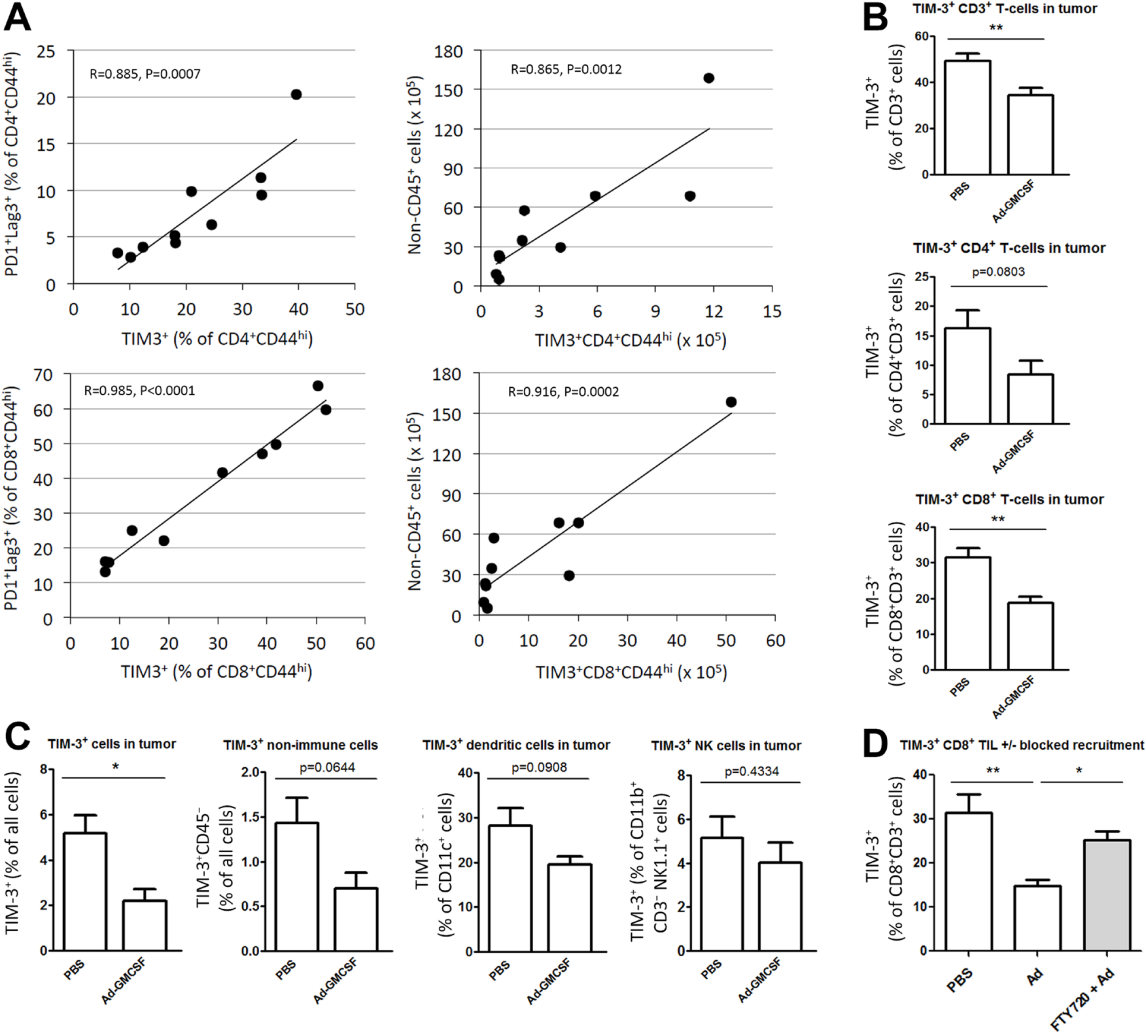
TIM-3 expression is associated with exhaustion phenotype and melanoma progression, while oncolytic adenovirus reduces the proportion of TIM-3^+^ subset among CD8^+^ TILs. (A) Mice bearing 7-day established subcutaneous B16-OVA tumors were euthanized, and tumor-infiltrating cells were analyzed by flow cytometry. Exhaustion-associated marker TIM-3 on activated CD4^+^ (top) and CD8^+^ TIL (bottom) as a function of highly exhausted PD-1^+^ and Lag-3^+^ subset by frequency (left), or as a function of total tumor cell burden indicating melanoma progression (right). (B–D) Mice bearing B16-OVA tumors were treated with Ad-GMCSF or Ad5/3-∆24 backbone virus (Ad) or PBS intratumorally and indicated tumor-infiltrating cells were analyzed by flow cytometry 12–14 days later. In addition, mice (D) received vehicle or FTY720 drug intraperitoneally starting 4 days before therapy onset, to block recruitment of new CD8+ TIL (see also online supplemental figure 1). Data represent individual tumors (A) and mean frequency (+SEM) (B–D) (n=4–5 per group). Pearson’s coefficient (A), unpaired t-test (B, C), one-way analysis of variance (D). *P<0.05, **P<0.01. Ad-GMCSF, Ad5/3-∆24-GMCSF virus; Lag-3, lymphocyte activation gene-3; PBS, phosphate-buffered saline; PD-1, programmed death-1; TIM-3, T-cell immunoglobulin and mucin domain-3.

Co-inhibitory receptor TIM-3 is upregulated on T-cell activation, but in concert with other immune checkpoints, remains elevated under chronic stimulation, thereby promoting T-cell exhaustion and dysfunction.^9^ We first studied the relation of TIM-3 to other established immune checkpoints on TILs in a mouse model of B16 melanoma. Cell surface expression of programmed death-1 (PD-1) and lymphocyte activation gene-3 (Lag-3) exhaustion markers strongly correlated with TIM-3 expression both on activated CD4^+^ (p=0.0007) and CD8^+^ TIL (p<0.0001, figure 1A). In addition, cell counts of CD4^+^TIM-3^+^ and CD8^+^TIM-3^+^ TIL linearly correlated with tumor cell burden (figure 1A), indicating that progressing B16 melanoma tumors accumulate exhausted TIM-3^+^ TIL that fail to control tumor growth.

We next examined the impact of oncolytic adenovirus on TIM-3 expression of several cell types in the tumor microenvironment. Fourteen days after intratumoral saline injections, the CD8^+^ TIL population showed a high frequency of TIM-3 expression (figure 1B, C). Remarkably, oncolytic adenovirus therapy reduced the proportion of TIM-3^+^ subset of CD8^+^ TIL by 68.1% on average (p=0.003, figure 1B). None of the other immune cells types were significantly impacted by virus treatment, despite CD4^+^ TIL and CD11c^+^ dendritic cells showing a similar trend (figure 1B, C). Intrerestingly, TIM-3 expression on non-immune cells also trended for decrease (figure 1C), although overall expression frequency remained low.

To further explore the mechanism behind TIM-3 decrease in CD8^+^ TIL, we conducted a similar experiment with intraperitoneal vehicle or FTY720 treatment, a compound inhibiting the recruitment of new lymphocytes into tumor. In the presence of FTY720, circulating lymphocytes were depleted (online supplemental figure S1A), and subsequent oncolytic adenovirus treatment failed to reduce TIM-3 levels on CD8^+^ TIL (figure 1D) while presenting reduced CD8^+^ TIL numbers (online supplemental figure S1B). These data indicate that the influx of new CD8^+^ T cells is crucial for the TIM-3 decrease, likely by diluting out exhausted subsets and/or remodeling the tumor microenvironment less conducive for inhibitory signals since also absolute TIM-3^+^ CD8^+^ TIL numbers trended lower (online supplemental figure S1C). We also tested PD-1 modulation, which showed a similar although lower degree of reduction on CD8^+^ TIL (online supplemental figure S1E). Protein-level reduction of TIM-3 on CD8^+^ TIL correlated with TIM-3 mRNA reduction in tumors after oncolytic adenovirus treatment (online supplemental figure S1E). Ratio of less-exhausted to terminally exhausted TIM-3^+^PD-1^hi^ CD8^+^ TIL was found highly elevated in oncolytic adenovirus-treated tumors on influx of new lymphocytes (online supplemental figure S1D), further supporting diminished exhaustion in tumors. Thus, active immunotherapy by oncolytic adenovirus alleviates the exhaustion phenotype and TIM-3 checkpoint expression on CD8^+^ TIL through recruitment of new lymphocytes into tumors. Taken together, our preclinical data reveal that TIM-3 expression on TIL associates with an exhausted phenotype and progressive disease in B16 melanoma, which can be counteracted by oncolytic adenovirus treatment.

### Patients with cancer experiencing TIM-3 downregulation in tumors following oncolytic adenovirus therapy show evidence of clinical activity and improved overall survival

**Figure 2:**
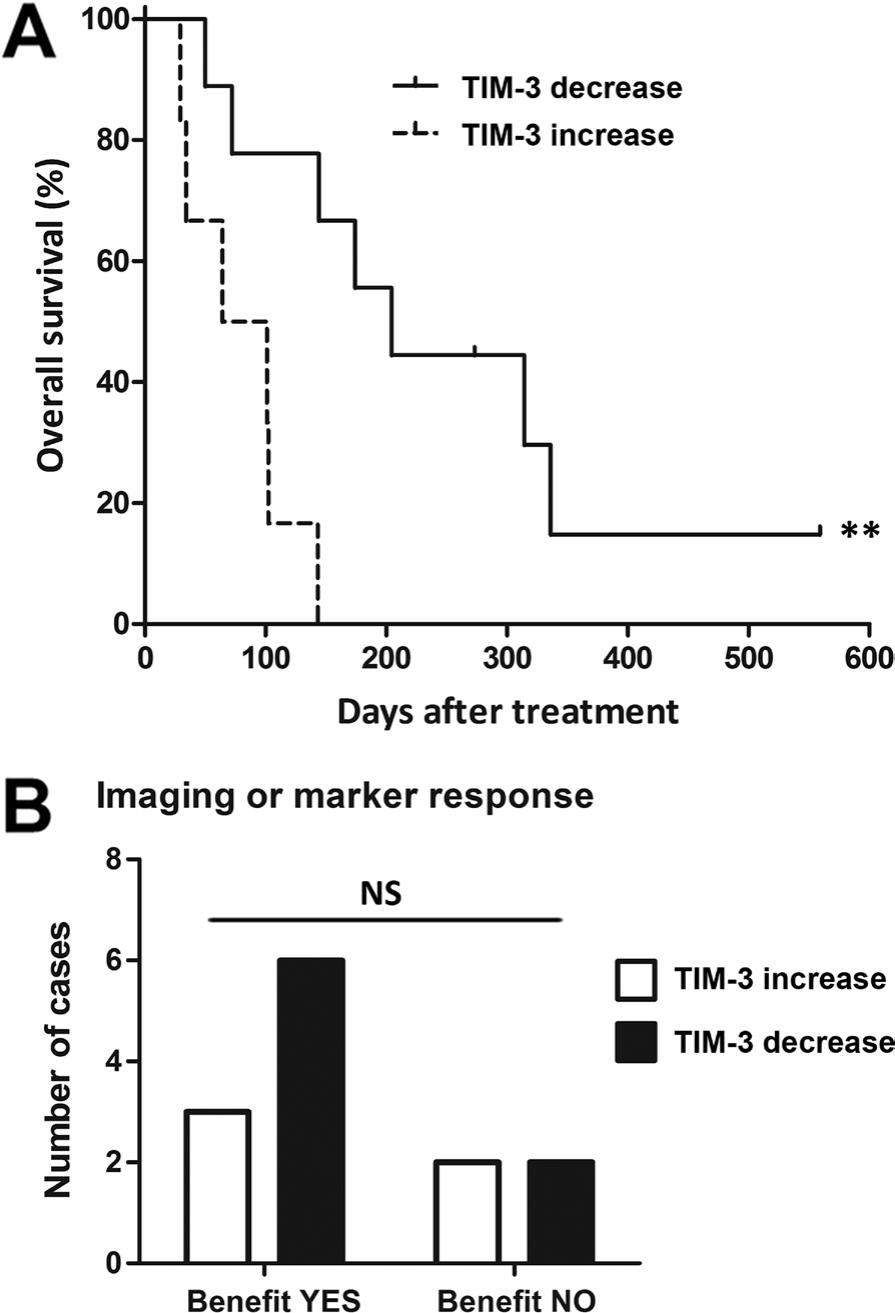
Patients with cancer experiencing decrease of TIM-3 expression in tumor site following oncolytic adenovirus therapy show improved overall survival. Patients with cancer were analyzed for tumor-site TIM-3 expression before and after the onset of oncolytic adenovirus therapy by microarray. (A) Patients with TIM-3 decrease presented a longer median overall survival of 204 days (95% CI 116 to 292 days) as compared to 64 days (95% CI 0 to 144 days) in patients with TIM-3 increase (p=0.0036, log-rank test). (B) Pretreatment and post-treatment PET-CT and tumor marker responses were scored by PET criteria or blood test described in online supplemental table S1; 75% of patients with TIM-3 decrease showed evidence of clinical benefit (disease stabilization or better), while 50% of patients with TIM-3 increase presented clinical benefit (Fisher’s exact test). **P<0.01. ns, not significant; PET, positron emission tomography; TIM-3, T-cell immunoglobulin and mucin domain-3.

Our preclinical findings encouraged us to conduct a longitudinal retrospective analysis of TIM-3 in patients with cancer undergoing oncolytic adenovirus therapy. We analyzed tumor TIM-3 expression change in 15 patients with advanced solid tumors refractory to conventional treatment (table 1). Microarray analysis of pretreatment and post-treatment pairs of tumor biopsy (or liquid biopsy of tumor-associated ascites/pleural effusion), obtained the day before and average of 8 weeks after oncolytic immunotherapy, revealed decrease of TIM-3 expression in nine patients, whereas six patients displayed increase of TIM-3 (online supplemental table S1). When assessing treatment benefit in these patients, we observed a significant survival benefit for patients experiencing TIM-3 decrease (median OS 204 days vs 64 days, p=0.0036; figure 2A). Although the relatively small cohort precluded conduction of multivariate analyses, the characteristics, sample material, or treatments did not differ between the groups (table 1), suggesting dynamic biomarker value for TIM-3 expression change during oncolytic adenovirus therapy.

For evaluation of treatment responses, pretreatment and post-treatment imaging data were available for 10 patients, and blood tumor marker responses could be evaluated in three additional cases (online supplemental table S1): evidence of clinical benefit (disease stabilization or better, in patients progressing prior to therapy) was observed in 75% of patients showing TIM-3 decrease, whereas 50% of patients with TIM-3 increase achieved clinical benefit (figure 2B). Notably, three patients in the TIM-3 decrease group had imaging responses (scored as minor response) as compared with only one patient in the TIM-3 increase group (table 2). In this relatively small cohort, however, the imaging/marker responses did not reach statistical significance (table 2), which may also in part reflect the reported difficulty of evaluating immunotherapy responses due to initial tumor swelling, that is, pseudo-progression, and accumulation of glucose-avid lymphocytes.^7 22^

**Table 2:** Treatment outcome after oncolytic adenovirus treatments in TIM-3 groups

Clinical parameter		TIM-3 decr. (n=9)	TIM-3 incr. (n=6)	Total (n=15)	Significance* (decr. vs incr.)	
Treatment outcome		Patient, n (% of total)			P value	
Marker response†	CR	1 (11.1)	0 (0.0)	1 (6.7)	ns	
	PR	0 (0.0)	2 (33.3)	2 (13.3)	ns	
	MR	2 (22.2)	0 (0.0)	2 (13.3)	ns	
	SD	2 (22.2)	0 (0.0)	2 (13.3)	ns	
	PD	2 (22.2)	3 (50.0)	5 (33.3)	ns	
	N/A	2 (22.2)	1 (16.7)	3 (20.0)	ns	
Imaging response†	CR	0 (0.0)	0 (0.0)	0 (0.0)	ns	
	PR	0 (0.0)	0 (0.0)	0 (0.0)	ns	
	MR	3 (33.3)	1 (16.7)	4 (26.7)	ns	
	SD	2 (22.2)	1 (16.7)	3 (20.0)	ns	
	PD	2 (22.2)	1 (16.7)	3 (20.0)	ns	
	N/A	2 (22.2)	3 (50.0)	5 (33.3)	ns	
Overall survival	Median	204 days	64 days	143 days	‡	(0.0036)
	95% CI	(116 to 292 days)	(0 to 144 days)	(89 to 197 d)		

*Patients were stratified into TIM-3 decr. and TIM-3 incr. groups based on direction of TIM-3 expression change. Fisher’s exact test was used for categorical response variables between TIM-3 groups, while log-rank test was used for overall survival.

†PET criteria were used to assess results of PET-CT imaging and tumor markers were measured from blood samples, both scored with the same percentage cutoffs, as described in the Materials and Methods section and online supplemental table S1.

‡P<0.01.

CR, complete response; decr., decrease; incr., increase; MR, minor response; ns, not significant; PD, progressive disease; PET, positron emission tomography; PR, partial response; SD, stable disease; TIM-3, T-cell immunoglobulin and mucin domain-3.

Taken together, decrease of the TIM-3 immune checkpoint on oncolytic adenovirus treatment was observed in 9 out of 15 patients, which was associated with significantly improved overall survival. Thus, expanding from our preclinical data, these results suggest favorable modulation of the tumor microenvironment by oncolytic adenovirus. Importantly, TIM-3 expression change at the tumor thus represents a potential novel biomarker for patients undergoing oncolytic adenovirus therapy.

### TIM-3 decrease after oncolytic adenovirus treatment is associated with reduction in its ligands and collective decrease in other T-cell coinhibitory receptors

**Figure 3:**
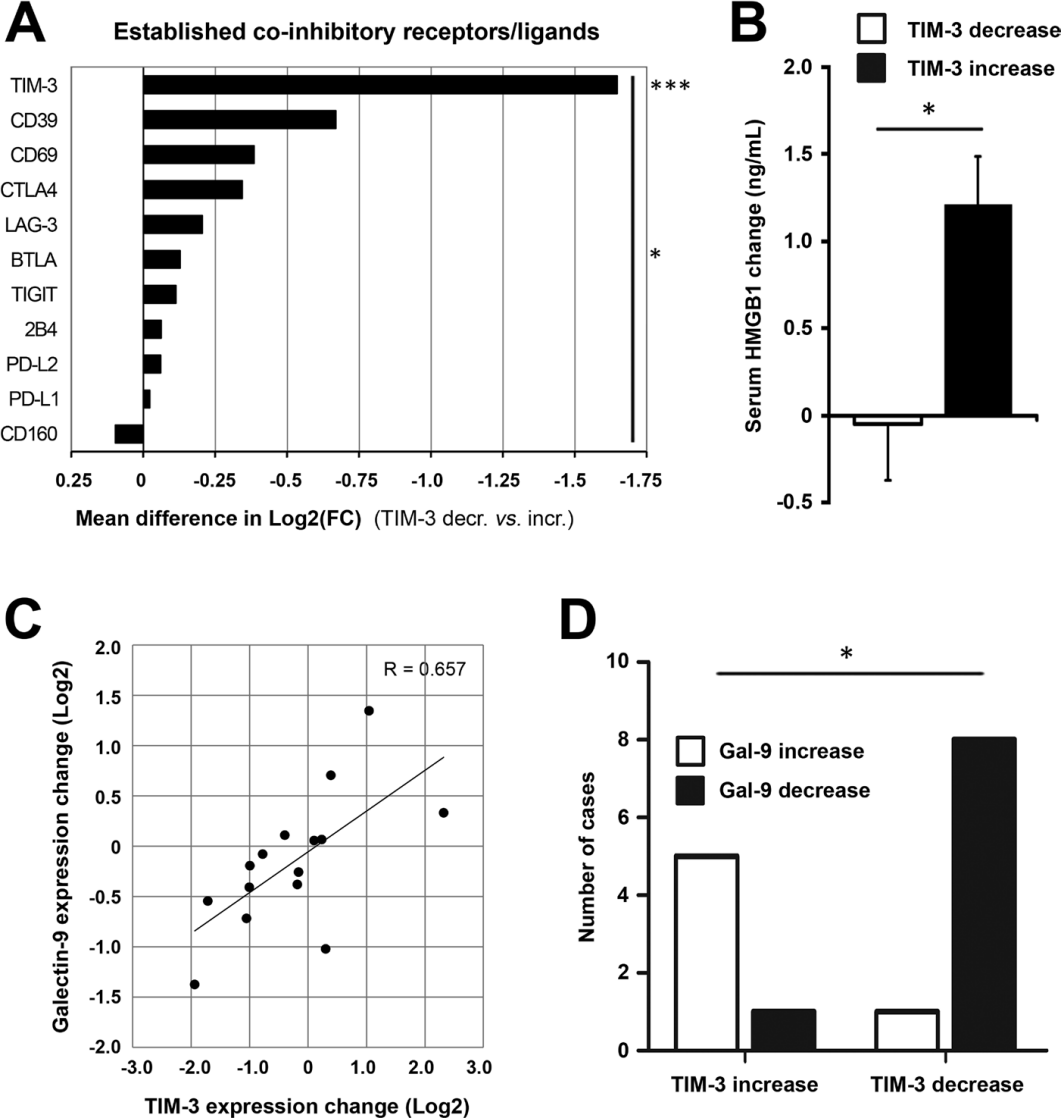
TIM-3 decrease after oncolytic adenovirus treatment is associated with reduction in its ligands and coinhibitory T-cell receptors. Coinhibitory receptors and ligands were explored in tumor-site expression data and patient serum. (A) Patients experiencing TIM-3 decrease showed significantly reduced expression of TIM-3 as compared with the increase group (−0.733 vs +0.914 mean log2(FC), respectively; p=0.0008) and collective reduction of coinhibitory receptors/ligands (p=0.011, comparing mean log2(FC) values between groups). Due to a technical defect, the PD-1 probe was not evaluable on the chip, but PD-L1 and PD-L2 ligands were studied instead. (B) Circulating HMGB1 protein, a ligand for TIM-3 receptor, showed an average reduction of −0.0492 ng/mL in TIM-3 decrease patients as compared with +1.209 ng/mL induction in TIM-3 increase patients (p=0.017). (C) Galectin-9 expression, another ligand for TIM-3 receptor, showed a linear correlation with TIM-3 expression change (Pearson’s R=0.657, p=0.0078). (D) Direction of galectin-9 change was associated with direction of TIM-3 change in 87% of cases (p=0.011). Unpaired t-test (A, B), Pearson’s coefficient (C); Fisher’s exact test in D. *P<0.05, ***P<0.001. HMGB1, high-mobility group box-1 protein; PD-1, PBS, phosphate-buffered saline; TIM-3, T-cell immunoglobulin and mucin domain-3.

We next studied the TIM-3 signaling pathway and related coinhibitory receptors. Patients experiencing TIM-3 decrease showed significantly reduced expression of TIM-3 as compared with the increase group (2.1-fold reduction vs 2.0-fold increase from baseline, respectively, p<0.0001; online supplemental table S2). Patients were then stratified according to the direction of TIM-3 change for further correlative analyses: 9 out of 10 immune checkpoints/ligands showed concomitant reduction in patients with TIM-3 decrease, which collectively was a significant difference (p=0.011, figure 3A). Thus, tumor site TIM-3 reduction after oncolytic adenovirus treatment was associated with a relative reduction in well-established T-cell coinhibitory receptors as well as ligands of PD-1. Modulation of TIM-3 was expectedly the only significant finding between TIM-3 groups (mean difference of 1.647 log2FC, p=0.0008; figure 3A), but it also emerged as the strongest, differentially regulated checkpoint/ligand when analyzed individually (online supplemental figure S2A). These findings further underscored the key role of TIM-3 signaling, as observed preclinically and across patient tumor types.^12^

After establishing TIM-3 modulation by oncolytic adenovirus as a potential marker of response, reflecting alleviation of exhaustion via TIL recruitment as observed preclinically, we focused on its signaling pathway. We measured circulating HMGB1 protein, a soluble ligand for TIM-3 receptor, and observed a concomitant change of −0.0492 and +1.209 ng/mL in TIM-3 decrease and increase patients, respectively (p=0.017, figure 3B), which also linearly correlated with TIM-3 change (p=0.0396, online supplemental table S2). Another TIM-3 ligand, galectin-9 (*LGALS9*), was studied pretreatment and post-treatment and showed a strong linear correlation with TIM-3 (p=0.0078, figure 3C and online supplemental table S2). Moreover, galectin-9 expression significantly differed between TIM-3 groups (online supplemental table S1), and its direction correlated with TIM-3 in 87% of cases (p=0.011, figure 3D). Other TIM-3 ligands, carcinoembryonic antigen cell adhesion-related molecule 1 (*CEACAM1*) and phosphatidylserine synthase (*PTDSS1*, not shown), which are ubiquitously expressed by tumor cells,^10^ showed overall reduction likely due to oncolysis and remodeling of the tumor microenvironment (online supplemental table S2). Notably, baseline levels of none of these ligands differed between study groups (online supplemental table S2). Finally, we surveyed downstream binding partners for human TIM-3, which showed concomitant reduction in the TIM-3 decrease group relative to the TIM-3 increase group (online supplemental figure S2B), with the exception of CD45, which is ubiquitously expressed by immune cells,^24^ thus collectively suggesting reduction of the immune inhibitory TIM-3 signaling cascade.

Taken together, galectin-9 and HMGB1, the two main protein ligands of TIM-3, correlated with TIM-3 expression and showed relative reduction together with downstream binding partners as well as other T-cell coinhibitory receptors in patients with TIM-3 decrease. These results indicate diminished activity of the suppressive TIM-3 signaling cascade, mediated potentially via recruited immune infiltrates as hinted in vivo, following oncolytic adenovirus treatment in patients with longer overall survival.

### Core transcriptional signature of CD8^+^ T-cell exhaustion is mitigated in virus-treated patients experiencing TIM-3 decrease

**Figure 4:**
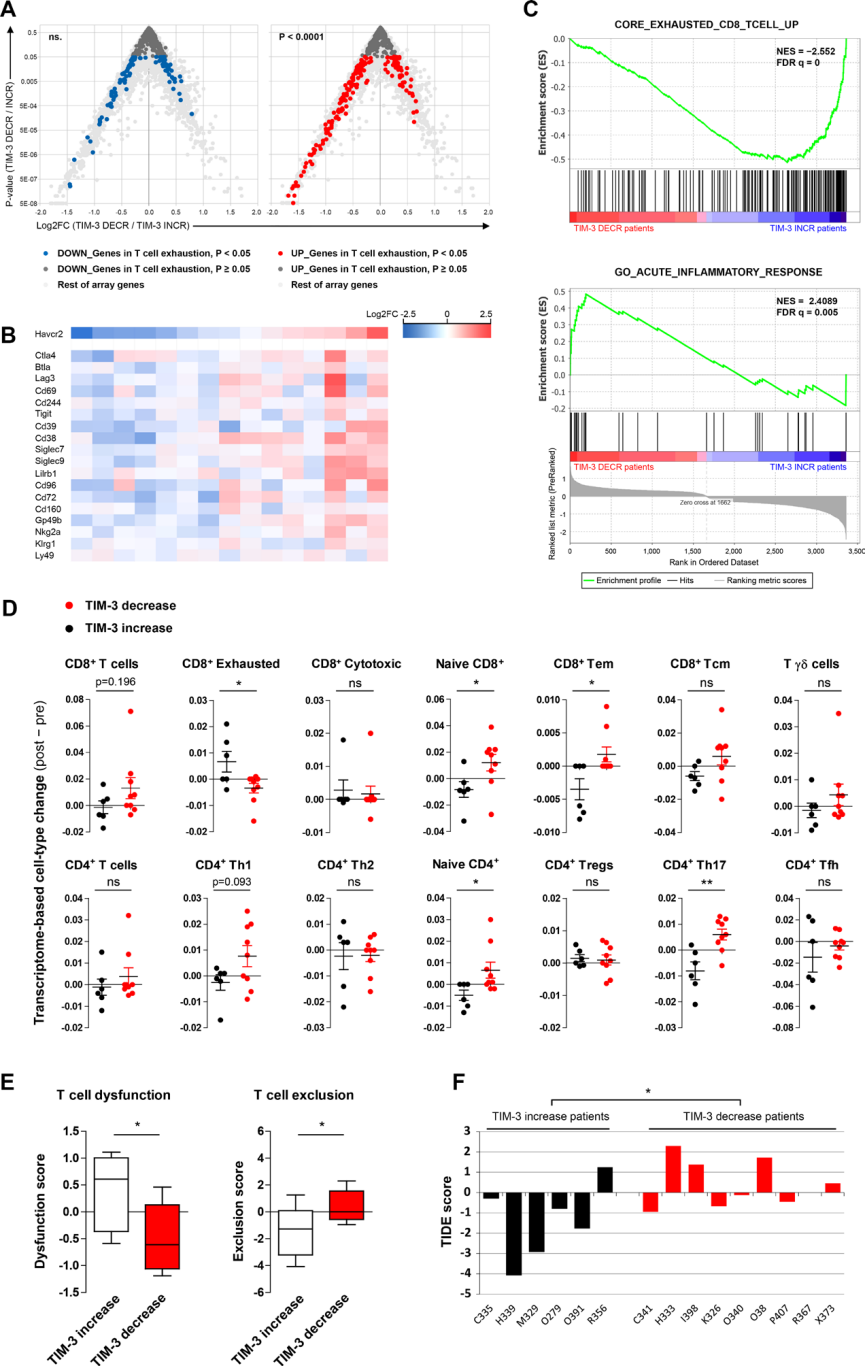
T-cell exhaustion and dysfunction are reduced in tumor site of TIM-3 decrease patients by oncolytic adenovirus therapy, while TIM-3 increase patients are predicted to potentially benefit from checkpoint blockade. (A) Volcano plots comparing transcriptomic changes in TIM-3 increase versus decrease patients following oncolytic adenovirus treatment showing genes that are either downregulated (left) or upregulated (right) in the core signature of CD8^+^ T-cell exhaustion: upregulated T-cell exhaustion genes were heavily skewed toward TIM-3 increase patients (p<0.0001), while downregulated T-cell exhaustion genes did not show enrichment in either group. (B) Heatmap of established and putative T-cell exhaustion markers in study patients sorted by TIM-3 modulation; blue indicates decreased and red increased expression. (C) GSEA of differentially regulated genes between TIM-3 decrease and increase patients (ranking metrics on the bottom). Top panel: Genes upregulated in the core exhausted CD8^+^ T-cell signature^25^ (normalized enrichment score −2.5523 toward TIM-3 increase group, false discovery rate-corrected p value of q<0.00001). Bottom panel: acute inflammatory response (GO:0002526, normalized enrichment score +2.4089 toward TIM-3 decrease group; *q*=0.00521). See also online supplemental figure S3 for other top GO terms. (D) Transcriptome-based cell-type quantification analysis by ImmuCellAI method,^35^ presented as individual abundance score change (post–pre) and compared between TIM-3 groups. (E, F) Preconditioning of tumor-site microenvironment for passive immunotherapy was analyzed by TIDE method^33^ surveying post versus pre virus therapy: T-cell dysfunction and T-cell exclusion scores of TIM-3 increase and decrease patients presented as whisker plots (E) and TIDE scores by patient to predict potential checkpoint blockade response (F, negative score=more likely to benefit from immune checkpoint blockade). χ^2^ test versus all differentially regulated genes (A), GSEA rank-based statistics (C), and unpaired t-test in (D–F). *P<0.05, **P<0.01; ns. FC, fold change; GSEA, gene set enrichment analysis; NES, normalized enrichment score; ns, not significant; TIDE, Tumor Immune Dysfunction and Exclusion; TIM-3, T-cell immunoglobulin and mucin domain-3.

Since TIM-3 decrease was associated with collective reduction of other T-cell coinhibitory receptors (figure 3A), we hypothesized that TIM-3 modulation by oncolytic adenovirus may reflect overall T-cell fate. Bengsch *et al* recently established the core transcriptional CD8^+^ T-cell exhaustion signature by combining mouse and patient expression data from chronic viral infections and cancer.^25^ We compared the transcriptomic changes of TIM-3 patient groups on oncolytic adenovirus therapy and observed a significant upregulation of the core T-cell exhaustion program in patients experiencing TIM-3 increase (p<0.0001, figure 4A). In line with this finding, several established and putative T-cell exhaustion markers showed associated changes with individual TIM-3 expression change per patient (figure 4B).

Immune-related gene signatures were further explored by gene set enrichment analysis (GSEA). Again, the core transcriptional signature of CD8^+^ T-cell exhaustion was strongly enriched in TIM-3 increase patients (q<0.00001, figure 4C). In striking contrast, TIM-3 decrease patients showed enrichment of acute inflammatory response (q=0.00521, figure 4C), when we ran the entire list of Atlas of Gene Ontology (GO) terms either by rank-based statistics or GSEA (online supplemental figure S3A). Bengsch *et al* reported the top 20 biological process GO terms among differentially expressed gene sets of exhausted CD8^+^ T cells.^25^ Many of the identical and related terms such as innate/chronic inflammatory response, chemotaxis, and cytokine biosynthesis were significantly upregulated in the TIM-3 increase group, similar to the core T-cell exhaustion (online supplemental figure S3A).^25^ Meanwhile, metabolic processes, acute inflammatory response, and protein activation cascade were associated significantly with TIM-3 decrease (online supplemental figure S3A), as with reduced T-cell exhaustion,^25^ indicating active immune response and mitigation of metabolic checkpoints.

In an attempt to characterize genes contributing to T-cell exhaustion, we performed leading edge analysis of three separate T-cell exhaustion/dysfunction signatures, each of which showed significant enrichment in the TIM-3 increase group (not shown), and studied their relationship to inflammatory and metabolic GO terms (online supplemental figure S3B). Potential drivers of T-cell exhaustion in TIM-3 increase patients included CD38 and CD39 (*ENTPD1*), which have been identified as exhaustion-specific molecules on human T cells,^26^ as well as calprotectin (*S100A9*) and fibrinogen-like protein-2 (*FGL2*), which have emerging roles in promoting T-cell exhaustion.^27–29^

Notably, TIM-3 (*HAVCR2*) was not part of any core signature of exhaustion/dysfunction, consistent with its dual role as an exhaustion and activation-associated marker expressed also transiently by effector cells.^10^ Nevertheless, induced TIM-3 expression at post-treatment biopsy contributed to several GO terms involved in immune regulation (online supplemental figure S3B). Several chemokine ligands and interleukin (IL)-1-beta (drivers of chemotaxis) and innate immunity-related terms were enriched only in TIM-3 increase patients, reflecting an inflamed tumor microenvironment.^30^ Meanwhile, acute inflammatory response genes *SAA1* and *ORM1/2* and metabolic enzymes *PHGDH* and *SHMT1/2*, which drive essential metabolite biosynthesis for effector T-cell expansion,^31^ were among the leading edge genes in TIM-3 decrease patients.

Taken together, oncolytic adenovirus therapy can lead to mitigation of T-cell exhaustion in tumors, supported by acute inflammatory responses and metabolic activity, particularly in a subset of patients who experience TIM-3 decrease. Moreover, these data reveal a previously uncharacterized link between oncolytic adenovirus-induced modulation of CD8^+^ T-cell exhaustion and survival benefit in patients, with notable implications for cancer immunotherapy.

### Cell-type enrichment analyses find T-cell dysfunction in TIM-3 increase patients and identify patients potentially benefiting from checkpoint blockade

Transcriptome-based cell-type quantification algorithms have been successfully applied to tumor immunology research,^32^ with emerging clinical value for immunotherapies.^33^ Since different tumor types exhibit variation in baseline cellular composition and gene expression levels (despite normalization and correction for batch effects),^32^ we concentrated on individual changes in cell-type composition. Global analysis of 64 cell types using the xCell method^34^ returned enrichment scores with changes such as reduction of epithelial (adenocarcinoma) cells and stromal components. These were likely due to tumor cell killing by oncolysis and effector-mediated cytolysis, as well as induction of granulocyte–monocyte and multipotent progenitor cells, which are typically associated with immunostimulatory GMCSF and CD40L transgenes^1 6^ (online supplemental figure S4). However, the greatest shift was revealed in the immune cell compartment: several myeloid and innate immune cell types showed reduction, whereas CD8^+^ and CD4^+^ T-cell subsets were the most enriched cell types.

Given the modulation of TIM-3 expression in the T-cell compartment in vivo (figure 1B), we further inquired CD8^+^ and CD4^+^ subsets by the Immune Cell Abundance Identifier (ImmuCellAI) method.^35^ While total CD8^+^ T cells trended for induction in TIM-3 decrease versus increase patients (p=0.196), the subset of exhausted CD8^+^ T cells was significantly reduced in the former group (p=0.022, figure 4D), further supporting mitigation of T-cell exhaustion. Effector-memory CD8^+^ T cells and Th17 cells were among the significantly induced subsets in TIM-3 decrease patients (figure 4D). Interestingly, also naïve-like CD8^+^ and CD4^+^ subsets were found preferentially induced in TIM-3 decrease patients, potentially reflecting epitope-spreading and mounting of de novo immune responses.

Oncolytic viruses are being evaluated in clinical trials in combination with checkpoint inhibitors with encouraging results.^4^ We therefore asked whether a subset of study patients might have benefited from this passive form of immunotherapy by conducting tumor immune dysfunction and exclusion (TIDE) analysis that compiles multiple published transcriptomic biomarkers and signatures to predict patient response to checkpoint blockade.^33^ In line with T-cell exhaustion, patients with TIM-3 increase showed significantly elevated T-cell dysfunction scores (p=0.033, figure 4E). Moreover, TIM-3 increase patients showed lower T-cell exclusion scores (p=0.036, figure 4E), indicating that inflamed ‘hot’ tumors are infiltrated by exhausted/dysfunctional T-cell clones. Indeed, the TIDE algorithm returned a predictive score for immune checkpoint blockade, which collectively would seem to predict possible benefit by checkpoint inhibition for TIM-3 increase patients: 83% of patients with TIM-3 increase showed a negative TIDE score as compared with only 44% in TIM-3 decrease group (figure 4F). By using a more stringent cut-off of −1 for stratifying patients, half of the patients experiencing TIM-3 increase and none of the patients with TIM-3 decrease were predicted to potentially benefit from immune checkpoint blockade (p=0.044, Fisher’s exact test; figure 4F).

### CD8^+^ T-cell redistribution and increased TIL infiltration are observed in patients with TIM-3 decrease, in association with prolonged survival

Recruitment of tumor-reactive CD8^+^ T-cell clones into tumors is the main goal of active immunotherapy. Transcriptome-based analyses suggested that TIM-3 decrease patients responded to active immunotherapy due to induction and remodeling of tumor-site immune subsets. To test this hypothesis, we performed immunohistochemistry on pretreatment and post-treatment biopsy samples of eight patients with available microarray data, and three additional patients receiving oncolytic virus therapy as an extension cohort (online supplemental table S1). Concentrating on the main transcriptome-based immune cell types modulated by oncolytic adenovirus therapy (online supplemental figure S4), myeloid and antigen-presenting populations (monocytes and macrophages, dendritic and B cells) showed only minor differences in immunohistochemistry, whereas the greatest impact was observed on CD8^+^ T lymphocytes (online supplemental figure S5A). Overall, 8 out of 11 patients showed evidence of increased CD8^+^ TIL infiltration following oncolytic immunotherapy.

**Figure 5:**
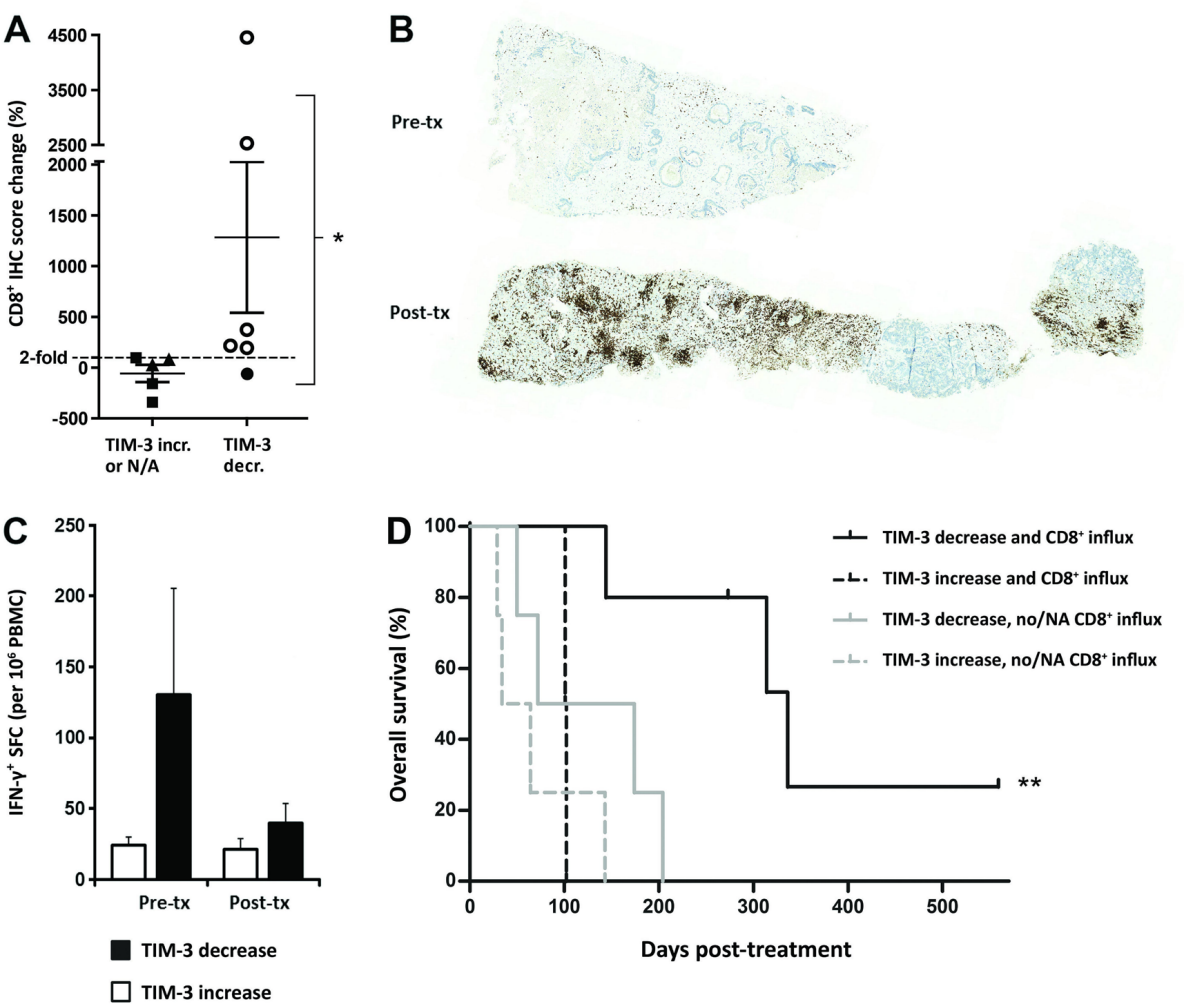
Increased CD8^+^ T-cell infiltration into tumors after oncolytic immunotherapy is observed in patients with concomitant TIM-3 decrease, which accounts for further improved overall survival. (A) Percentual change in CD8a IHC score following oncolytic adenovirus treatment in six patients with TIM-3 decrease (circle symbols) and five control patients (TIM-3 increase, triangle symbols; TIM-3 data not available, square symbols): ≥2 fold CD8^+^ increase was only observed in patients with concomitant TIM-3 decrease (open circles; p=0.015, Fisher’s exact test). (B) Representative CD8a IHC of liver metastasis biopsies of a colon adenocarcinoma patient C341 before (top) and after oncolytic adenovirus treatment (bottom) presenting a 45-fold increase (+4454.1%) in CD8a^+^ IHC score (brown color). (C) PBMCs collected on the same days as microarray samples were assessed for tumor-associated antigen (survivin) reactive effector cells by IFN-γ ELISPOT. TIM-3 decrease patients showed high baseline effector responses in blood, followed by reduction in circulation and concomitant CD8^+^ TIL increase in tumors on oncolytic virus therapy (A, B), compatible with CD8^+^ T-cell trafficking. Limited numbers of subjects had available PBMC samples (n=6 and n=3 in TIM-3 decrease and increase groups, respectively), which precluded statistical determination. See also online supplemental figure S6 for linear correlation of CD8^+^ redistribution compatible with trafficking phenomenon. (D) Kaplan-Meier analysis of subgroups based on both TIM-3 status and evidence of CD8^+^ influx into tumors: TIM-3 decrease patients who experienced CD8^+^ T-cell influx (open circles in A) survived longer than patients without both of these phenomena (median OS 336 days vs 72 days in combined others, p=0.002, log-rank test). See also online supplemental figure S7 for survival analysis based also on *CD8A* expression data in patients that lacked CD8a immunohistochemistry data (NA). *P<0.05, **P<0.01. IFN-γ, interferon gamma; IHC, immunohistochemistry; N/A, not available; OS, overall survival; PBMC, peripheral blood mononuclear cell; SFC, spot-forming colonies; TIM-3, T-cell immunoglobulin and mucin domain-3.

TIM-3 decrease patients had significantly higher increases of CD8^+^ TIL over control patients (p=0.044, online supplemental figure S5B). This was attributed to differential CD8^+^ TIL induction post-treatment as baseline CD8^+^ immunohistochemistry scores did not differ (online supplemental figure S5B). Particularly, twofold or greater CD8^+^ TIL influx was only observed in patients with confirmed tumor-site TIM-3 decrease (p=0.015, figure 5A), consistent with the TIM-3 checkpoint limiting CD8^+^ T-cell responses. Remarkably, two patients with TIM-3 decrease experienced very strong CD8^+^ influxes with immunohistochemistry scores increasing over 25-fold (figure 5A). Colon adenocarcinoma patient C341, who benefited from virus treatment by disease stabilization (both imaging and tumor marker evaluation), showed the greatest increase of +4454% in CD8^+^ T-cell infiltration, essentially turning ‘cold’ pretreatment tumor areas into heavily CD8^+^ infiltrated hot tumors (figure 5B). Ovarian adenocarcinoma patient O341, who experienced a minor response by imaging and a complete response by tumor marker evaluation together with ongoing survival of 559 days, likewise presented a robust +2532% influx of CD8^+^ T cells into the tumor bed.

We next analyzed effector cell activity in circulating blood by IFN-γ ELISPOT assay against a ubiquitous tumor-antigen survivin, which mainly comprises T-cell responses.^5^ Notably, IFN-γ responses were assessed without prior stimulation or expansion ex vivo, and thus the counts represent actual numbers present in blood. Patients with TIM-3 decrease showed a high average effector cell activity of 130.6 spot-forming colonies in blood before treatment as compared with 24.4 spot-forming colonies in patients with TIM-3 increase (figure 5C). However, peripheral blood effector cell activity after oncolytic virus treatment reduced to equal levels. Patients with TIM-3 decrease thus appeared to have tumor-reactive circulating T-cell clones that were redistributed from blood presumably into tumors on oncolytic adenovirus therapy. In particular, patients C341 and O340, who both experienced TIM-3 decrease, showed concomitant reduction in circulating T-cell activity and robust CD8^+^ T-cell influx into tumors (online supplemental figure S6A). This phenomenon has been linked to trafficking of antitumor T cells into the tumor,^3^ as further supported by our data herein (figure 5A–C and online supplemental figure S6A).

Interestingly, overall CD8^+^ T-cell frequency in blood seemed to increase linearly with CD8^+^ TIL infiltration (online supplemental figure S6B). Patients C341 and O340 experienced notable +33.8% and +77.8% expansions in peripheral blood CD8^+^ population from baseline, respectively, while presenting the aforementioned +4454% and +2532% increases in CD8^+^ TIL infiltration into tumors. In contrast, patients with a lesser degree of CD8^+^ TIL infiltration did not show major circulating CD8^+^ changes (online supplemental figure S6B). Furthermore, expansion of the CD8^+^ population in blood associated with concomitant reduction/trafficking of IFN-γ producing cells from peripheral blood presumably into tumor tissues (online supplemental figure S6C). While these findings warrant studies in larger cohorts, one can speculate that they reflect mobilization of new CD8^+^ T-cell clones, that are possibly naïve-like and directed against other tumor-associated antigens beyond survivin, as indicated by our transcriptome-based analyses (figure 4D). Importantly, CD8^+^ immunohistochemistry data also correlated with, and confirmed the utility of, transcriptome-based CD8^+^ T-cell data (p=0.048, online supplemental figure S6D).

Finally, we explored whether the observed CD8^+^ TIL influx was associated with long-term benefits to patients. When TIM-3 patients were subgrouped according to CD8^+^ TIL infiltration change by immunohistochemistry, the median overall survival of patients experiencing both TIM-3 decrease and CD8^+^ influx increased significantly to 336 days as compared with 72 days in the other patients (p=0.002, figure 5D). Similar findings were observed whether CD8^+^ infiltration was explored by immunohistochemistry or also estimated by *CD8A* mRNA expression: each comparison revealed TIM-3 decrease coupled with CD8^+^ increase as the longest surviving subgroup (online supplemental figure S7). Interestingly, however, increased CD8^+^ infiltration alone, either by CD8a immunohistochemistry or also by mRNA expression, failed to separate survival benefit in this cohort (online supplemental figure S7).

Taken together, our immunological data indicate mobilization of CD8^+^ T cells, redistribution of tumor-reactive clones, and most importantly, prominent CD8^+^ T-cell influx into tumors of patients experiencing TIM-3 decrease, which was associated with the strongest survival benefit. Hence, our translational study uncovers that oncolytic adenoviral immunotherapy results in TIM-3 reduction and mitigation of T-cell exhaustion through induction of intratumoral CD8^+^ immune response, which correlates with improved overall survival in patients with cancer.

## Discussion

Cancer immunotherapy is transforming clinical oncology practice, offering hope even for patients with metastatic disease. However, given rising costs and risks for immune-related adverse events,^4^ biomarkers are urgently needed for identification of patients likely to benefit from different types of immuno-oncology, that is, active immunotherapy inducing de novo antitumor CD8^+^ responses versus passive immunotherapy reinvigorating dysfunctional antitumor immunity. Oncolytic adenoviruses represent a robust modality of active immunotherapy. Recently, baseline prognostic factors have been identified, with focus on inflammatory mediators and (antiviral) innate immunity.^13 14 36 37^ Our translational study herein addresses adaptive immunity, and spesifically modulation on treatment, which is the key to deciphering dynamic tumor-immune interactions. Eventually, our results may contribute to adoption of effective immunotherapies in patients conventionally considered ineligible or poorly responsive (immune desert tumors, few mutations, PD-L1 low).

In this study we report, for the first time, the capacity of oncolytic adenovirus to dampen the proportion of TIM-3 positivity on CD8^+^ TIL in vivo and characterize two subgroups of patients, defined by differential modulation of TIM-3 signaling. These groups differ in terms of CD8^+^ T-cell exhaustion and associated metabolic and inflammatory signatures, redistribution and influx of CD8^+^ T cells into tumors, and most importantly, by long-term outcome following oncolytic immunotherapy. Evidence of clinical benefit was observed at 75% and 50% rates, respectively, in TIM-3 decrease versus increase groups, although without statistical difference, highlighting the challenges of evaluating immunotherapy outcome by traditional methods.^22^ Meanwhile, median overall survival was more than triple, and was further increased to 4.7 times longer in TIM-3 decrease patients, when CD8^+^ TIL influx was considered. This finding represents the largest survival increase identified in the search for clinical and biological prognostic and predictive factors for oncolytic adenoviruses.^5 13 14 21 23 36 37^ Thus, TIM-3 modulation could serve as a dynamic biomarker predicting survival benefit, which warrants future studies in prospective clinical trials.

Indicating an active role for TIM-3 checkpoint signaling, the main ligands, galectin-9 and HMGB1, as well as downstream binding partners correlated with TIM-3 expression in patient tumors and serum. Thus, our data suggest that the ligands are associated with TIM-3 change in tumors, although we acknowledge that it does not prove ligand–receptor interaction, which should be assessed in future biochemical studies. We have previously shown that elevated baseline HMGB1 is a negative prognostic factor,^13^ whereas temporary HMGB1 increase and subsequent decrease are associated with redistribution of antitumor T cells and treatment benefit.^21^ Similarly, high TIM-3 baseline expression was identified as a negative prognostic factor,^14^ whereas patients experiencing TIM-3 decrease during therapy showed induction of an acute inflammatory process and CD8^+^ TIL infiltration. Thus, modulation of TIM-3 and its ligands such as HMGB1 is likely to reflect the same phenomena where an acute surge reflects T-cell activation and mobilization, whereas chronic exposure limits antitumor immune responses. In other words, chronically inflamed hot tumors may not require active immunotherapy, but instead single or combined checkpoint inhibition.

While CD8^+^ TIL infiltration is generally a favorable prognostic factor per se,^9^ certain T-cell subsets have greater capacity to mediate antitumor responses.^38^ Our data revealed overall increase of CD8^+^ TIL among TIM-3 decrease patients, and particularly of naïve-like and effector memory subsets. Naïve-like CD8^+^ found in human tumors can evoke IFN-γ responses^39–41^ and is a useful source for adoptive cell therapy in vivo due to stemness and extended viability,^40^ while effector-memory CD8^+^ can produce effector cytokines and eradicate established tumors.^38^ We also observed induction of Th17 cells, which, despite controversy around their role in cancer, have been indicated to enhance long-term antitumor immunity.^42^ Th17 cells could also give rise to IFN-γ-secreting Th1 cells,^43^ which trended for induction as well (p=0.093). Most notably, induction of these subset signatures coincided with reduction in exhausted CD8^+^ T cells, indicating beneficial remodeling of the tumor microenvironment in TIM-3 decrease patients.

Our preclinical findings pinpoint the critical role for newly recruited lymphocytes in mediating decrease in TIM-3 and potentially its ligands on successful active immunotherapy, likely via dilution effect, although the CD8^+^ TIL infiltrate may also contribute to TIM-3 signaling modulation per se via cytokine production and remodeling of tumor microenvironment.^10^ While our study is the first to report mitigation of TIM-3^+^ population and T-cell exhaustion by an oncolytic adenovirus, through recruitment of new CD8^+^ T cell clones, Feist *et al* reached a similar conclusion for an oncolytic vaccinia virus that decreased the proportion of exhausted PD-1^hi^TIM-3^+^ subset within the expanded total CD8^+^ infiltrate,^17^ thus suggesting similar effects for other oncolytic viruses as well. We identified several putative drivers (eg, CD38, CD39, S100A9, and FGL2) of T-cell exhaustion/dysfunction in TIM-3 increase patients. Among them, both TIM-3^+^CD39^+^ and TIM-3^+^CD38^+^ phenotypes have been associated with CD8^+^ T-cell dysfunction in patients,^44 45^ contributing to suppressive purinergic signaling and metabolic perturbations.^46^ Indeed, metabolic arrest of T cells is regarded as a major driver of T-cell exhaustion.^9^ Further suggesting therapeutic value, severely exhausted TIM-3^+^CD39^+^ TIL were responsive to dual checkpoint blockade that improved survival in mice,^45^ much as predicted by TIDE analysis in our study (figure 4E, F). Regarding virus transgenes used as immune adjuvants, certain cytokines such as IL-2 may affect the exhaustion phenotype directly,^17^ whereas the widely used GMCSF or CD40L mainly act on immune cell recruitment and the priming phase.^1 6^ Indeed, our preclinical analyses showed that also the backbone virus without transgene resulted in decreased TIM-3 positivity.

Interestingly, also type I interferon response genes such as myxovirus-resistance proteins *MX1/2* and interferon-induced proteins *IFI-27* and *IFI-44* were among the leading edge genes of T-cell exhaustion. Besides a key role in innate immunity contributing to oncolytic adenovirus resistance,^47 48^ recent data indicate that type I interferon response appears as a major driver of coinhibitory receptor expression in human T cells.^49^ Intriguingly, also TIM-3 was found upregulated by interferon-β signaling,^49^ corroborating our previous and current studies of predictive and prognostic impact of MXA and TIM-3 during oncolytic virus therapy.^14^

Supporting clinical utility, our findings also support combination immunotherapy particularly for TIM-3 increase patients. Several preclinical studies suggest additive or synergistic efficacy for combination of oncolytic viruses and checkpoint blockade.^3 4^ Meanwhile, TIM-3 blockade has been suggested to synergize preclinically with dual blockade and active forms of immunotherapy as well.^10^ As all our study patients were initially immunotherapy naïve, and a typical timeline in clinical trials includes oncolytic virus treatment before onset of checkpoint blockade,^4^ the post-treatment biopsies collected at an average of 8 weeks would serve as an immune landscape ‘primed’ by oncolytic virus for checkpoint inhibition. Indeed, one of the most promising clinical data includes intratumoral injections of an oncolytic virus (talimogene laherparepvec) for 6 weeks, followed by the onset of checkpoint blockade pembrolizumab.^50^ Therefore, identification of patients (before the onset of checkpoint blockade) that are likely to achieve benefit from combination therapy versus oncolytic virus monotherapy would be valuable in order to reduce the costs and suffering due to side-effects of checkpoint blockade. Our transcriptome-based analyses identified both T-cell exhaustion and dysfunction signatures, cell-type enrichments that correlated with CD8^+^ immunohistochemistry, and possibly predicted responsiveness to checkpoint blockade (TIDE analysis). While the latter data are compelling, they are hypothesis-generating at this stage and should be validated in prospective clinical trials.

Although heterogeneity of the patient population may confound tumor-type spesific interpretation, immune system-related pathways are regarded universal, and findings obtained in a mixed solid tumor population may be more generalizable.^12^ We obtained histologically verified pretreatment and post-treatment biopsies from the same tumor location where oncolytic virus injections were administered. Thus, our longitudinal study design eliminates potential confounding factors related to interpatient and intrapatient variation, and allows for identification of immune cell changes—the target cell compartment for immunotherapies.^32^ Reflecting global modulation of tumor–immune landscape, stratification to TIM-3 groups separated phenotypes based on collective regulation of immune checkpoints, TIL infiltration, and antitumor immunity.^33^ Of note, future studies should assess the role of PD-1, which was not evaluable in patient microarrays due to technical defect but appeared as another candidate marker of therapy outcome in our preclinical data. Collectively, our results suggest utility for data integration (microarray/RNAseq, functional immunoassays, and immunohistochemistry) and computational modeling tools to refine biomarkers and potentially identify responders to combination immunotherapies, which is particularly valuable for a multimechanistic immunotherapeutic modality such as cytokine-armed oncolytic virus.

In conclusion, our results indicate that oncolytic adenovirus treatment can dampen the suppressive TIM-3 checkpoint pathway in murine and patient tumor microenvironments through recruitment of new CD8^+^ TIL, which reflects alleviation of T-cell exhaustion and adaptive immune modulation across tumor types and correlates with improved overall survival. Our findings also provide potential targets and strategies for extending the utility of immunotherapy to larger patient groups. Importantly, our study identifies TIM-3 modulation over time as a potential predictive biomarker for active immunotherapy by oncolytic adenoviruses, stratifying patients into those benefiting from oncolytic adenovirus monotherapy or potentially from single or combined checkpoint blockade. Prospective studies exploring the impact of the TIM-3 pathway on oncolytic immunotherapy and its predictive value as a dynamic biomarker are warranted.

## Data Availability

Data are available upon reasonable request. All data relevant to the study are included in the article or uploaded as supplementary information. Preclinical and deidentified subject (microarray, biological and clinical) data will be shared, upon a reasonable proposal approved by an independent review committee. Proposals should be sent to the corresponding author.
