# Supplementary material for: Oncolytic adenovirus decreases the proportion of TIM-3+ subset of tumor-infiltrating CD8+ T cells with correlation to improved survival in patients with cancer
Source: J Immunother Cancer. 2022 Feb 22;10(2):e003490. doi: 10.1136/jitc-2021-003490 (PMC8867324; doi:10.1136/jitc-2021-003490)
Supplement: Supplementary data [file jitc-2021-003490supp001.pdf]

Supplemental Material

Article title: Oncolytic adenovirus decreases the proportion of TIM-3$^+$ subset of tumor-infiltrating CD8$^+$ T cells with correlation to improved survival in cancer patients

Authors: Ilkka Liikanen, Saru Basnet, Dafne C. A. Quixabeira, Kristian Taipale, Otto Hemminki, Minna Oksanen, Matti Kankainen, Juuso Juhila, Anna Kanerva, Timo Joensuu, Siri Tähtinen, Akseli Hemminki

The Supplemental Material includes:

Supplementary Figures S1-7

Supplementary Tables S1-2

Supplementary Materials and Methods

Corresponding author: Akseli Hemminki (akseli.hemminki@helsinki.fi)

Supplementary Figures and legends

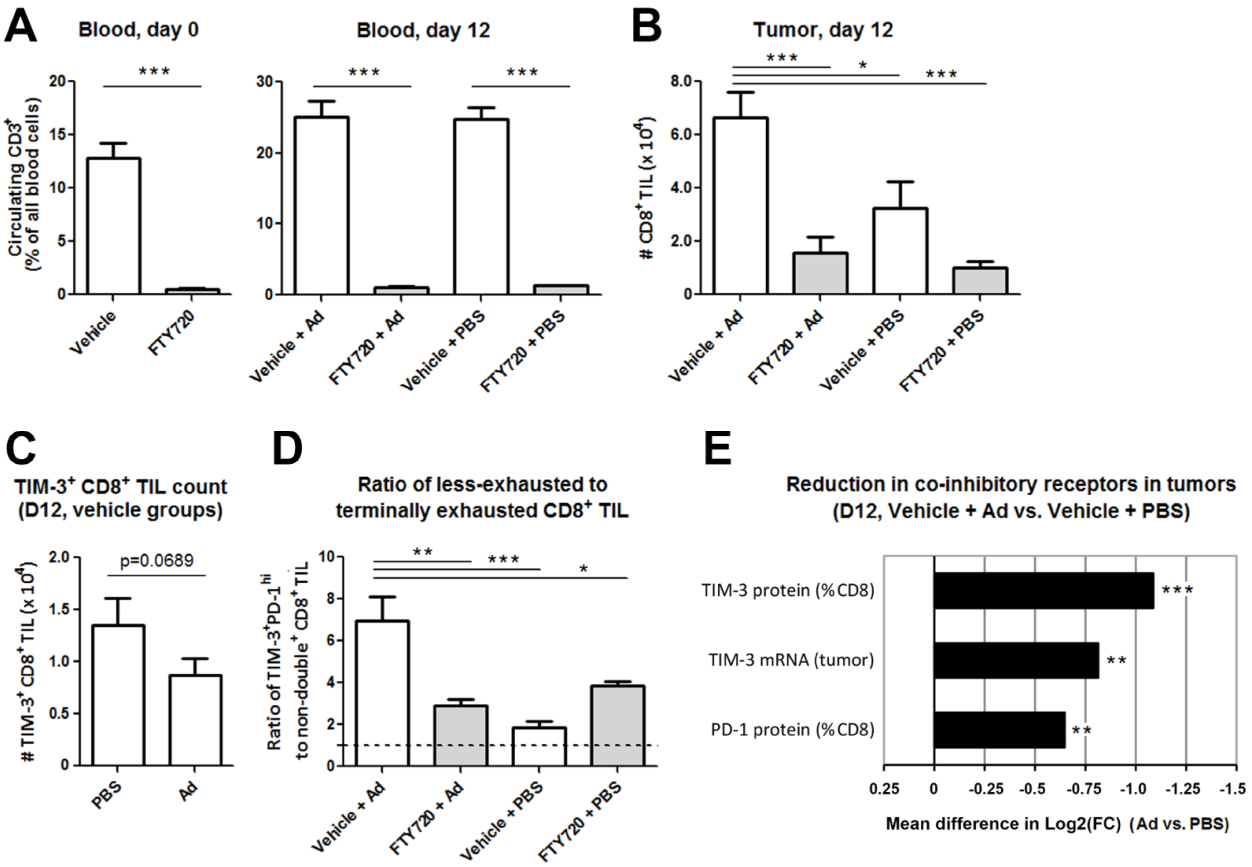

Fig. S1 Liikanen et al.

Supplementary Figure S1: Oncolytic adenovirus treatment results in reduced proportional TIM-3$^+$ and PD-1$^+$ TIL subsets along with decreased TIM-3 mRNA levels in tumors, due to recruitment of new CD8$^+$ T cells via circulation.

A-D) B16-OVA tumor-bearing mice received daily intraperitoneal injections of 1 mg/kg of FTY720 or vehicle (1.3% DMSO in PBS) starting 4 days before, continuing during the six consecutive intratumoral treatment days with oncolytic adenovirus Ad5/3-Δ24 (Ad) or mock treatment (PBS), and continuously thereafter. **A)** Mice were bled from lateral saphenous vein before (day 0) and 12 days after oncolytic virus therapy onset, and circulating CD3$^+$ T lymphocytes were analyzed by flow cytometry to confirm the effect of FTY720 drug (grey bars) in inhibiting lymphocyte recruitment via circulation. **B-D)** Mice were

euthanized on day 12 post-treatment and tumor-infiltrating cells were counted and analyzed by flow cytometry: Absolute numbers of $CD8^+$ TIL in end-point tumors showed significant induction after oncolytic adenovirus treatment, but reduction in FTY720 treated animals reflecting impaired TIL recruitment (**B**). Absolute TIM-3^+ $CD8^+$ TIL were further counted which trended for lower numbers following oncolytic adenovirus treatment (P=0.0689; in **C**), despite the higher overall $CD8^+$ TIL numbers as shown in (**B**). Terminally exhausted $CD8^+$ TIL co-expressing TIM-3^+ PD-1^{hi} were counted based on multi-color flow cytometry data, and their relative abundance to less-exhausted counterparts (not double-positive) were surveyed: Oncolytic adenovirus treatment resulted in 6.9 times higher levels of less-exhausted over terminally exhausted subsets, whereas mock-treated animals showed only ratio of 1.9 (P=0.0004 *vs*. Ad-treated) similar to FTY720 treated groups with significantly lower ratios as well. Dotted line indicates ratio of 1.0 (= equal numbers of less-exhausted to terminally exhausted TIL). **E)** To compare relative reduction between whole tumor mRNA levels of TIM-3 (*HAVCR2* gene transcript) and protein levels of PD-1 and TIM-3, end-point tumors were analyzed by both RT-qPCR of the extracted total mRNA and by flow cytometry of $CD8^+$ TIL: mRNA transcript levels of TIM-3 showed significantly decreased levels (P=0.0064, Ad-treated vs. mock-treated), corresponding to the significantly reduced proportion of TIM-3 protein expression on $CD8^+$ TIL (P=0.0008, Ad-treated vs. mock-treated). Likewise, PD-1 protein showed also concomitant reduction (P=0.0071, Ad-treated vs. mock-treated), although its relative reduction on $CD8^+$ TIL was 1.7 times lower than for TIM-3 protein (mean -1.087 log2FC for TIM-3 vs. -0.647 log2FC for PD-1). Data represent mean frequency/count (+SEM) in B-D and mean reduction in E (n=4-5 per group). *, P<0.05; **, P<0.01; ***, P<0.001; Unpaired t-test in A, C, E; One-way ANOVA in B and D.

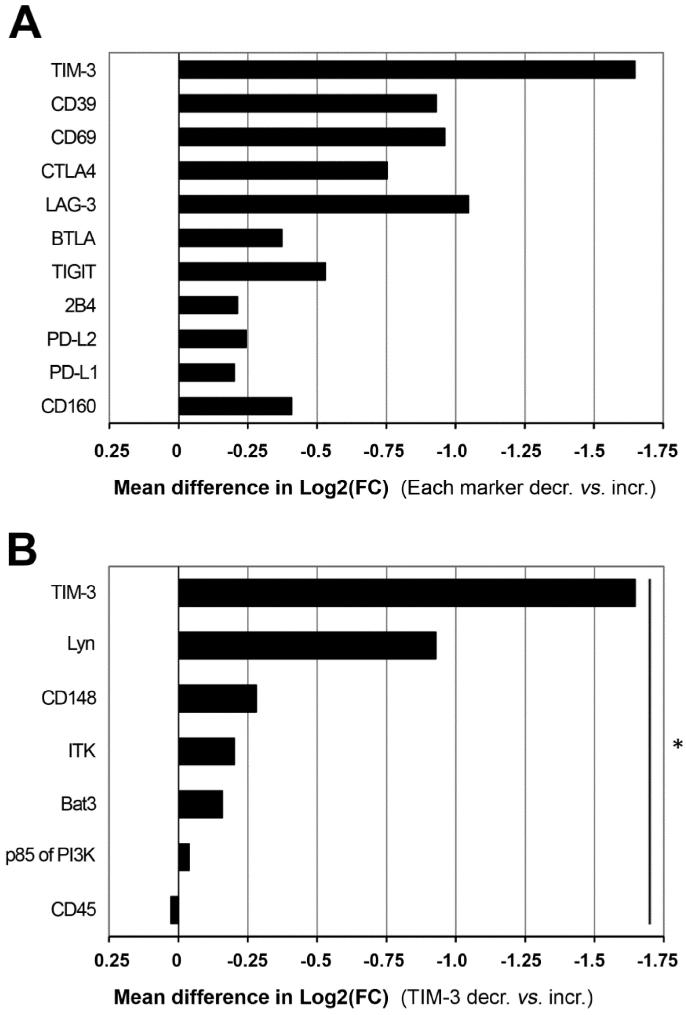

Fig. S2 Liikanen et al.

Supplementary Figure S2: TIM-3 shows the most differentially regulated expression of established exhaustion-associated markers, and its downstream binding partners correlate with TIM-3 decrease.

A) Established co-inhibitory markers were explored in tumor-site expression data as in figure 3A, but were stratified based on each individual marker change: Difference in average logarithmic fold change between patients with decrease *versus* increase of the particular marker are shown. Cross-comparison between individual markers revealed that TIM-3 modulation emerged as the most differentially regulated marker with 1.6 times stronger modulation than the LAG-3 marker (-1.647 log2[FC] in TIM-3 *versus* -1.047 log2[FC] in LAG-3), and 2.9 times stronger than the mean modulation of other markers (-1.647 log2[FC] in

TIM-3 *versus* average of -0.565 log2[FC] in others). **B)** Downstream binding partners for human TIM-3 [20] were surveyed in tumor-site expression data and their relative change was compared between TIM-3 decrease versus increase patients: Difference in average logarithmic fold change (TIM-3 decr. vs. incr.) is shown for each transcript, which showed collectively a significant reduction in TIM-3 decrease over TIM-3 increase patients (P=0.027), suggesting reduction of the inhibitory TIM-3 signaling cascade. Of note, CD45 has a dual role in regulating T cell receptor signaling and is ubiquitously expressed by immune cells, consistent with the minor increase observed in TIM-3 decrease patients that presented influx of TIL (see figures 5 and S7).

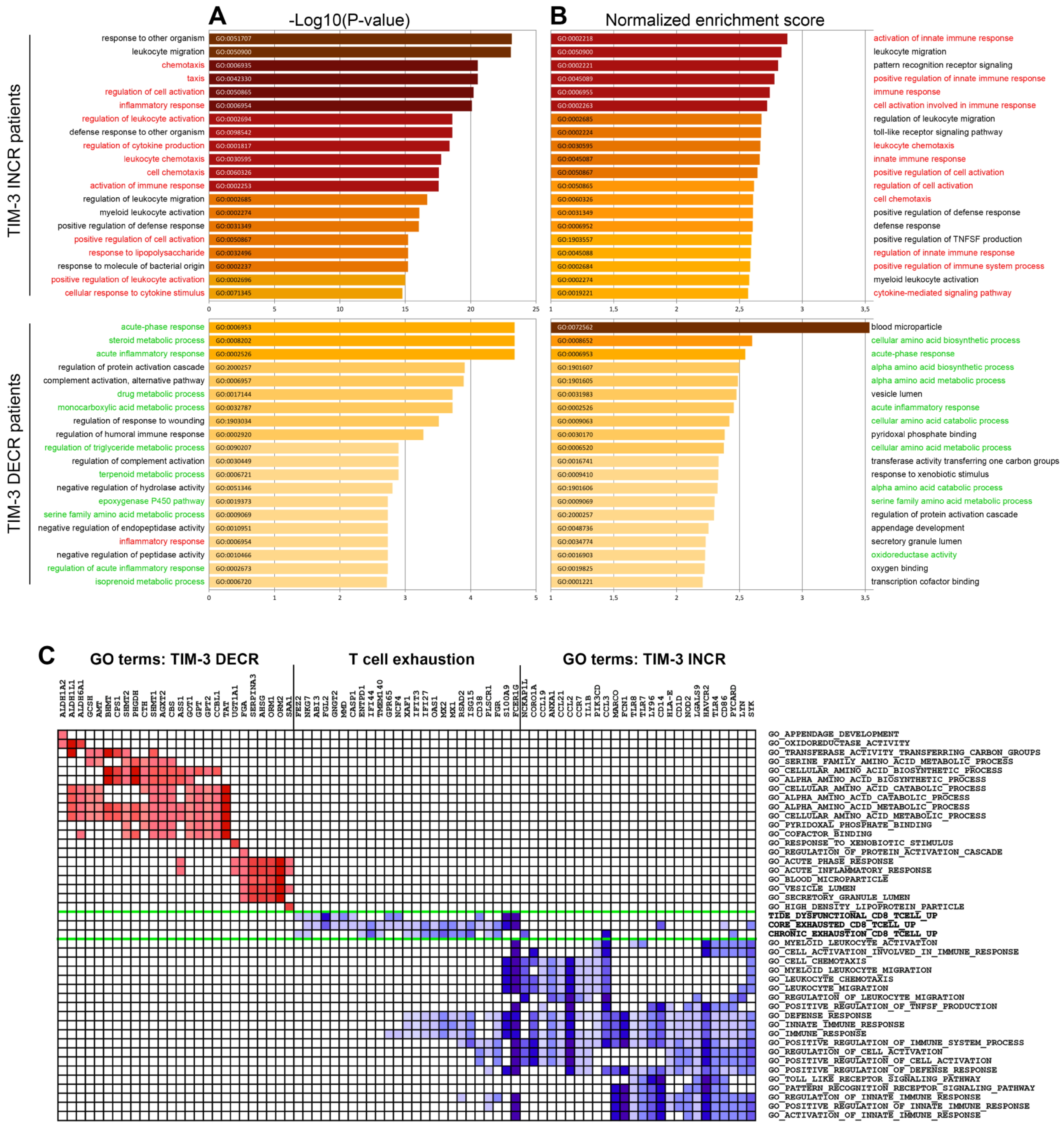

Fig. S3 Liikanen et al.

Supplementary Figure S3: Enriched Gene Ontology (GO) terms between TIM-3 groups are not redundant, coherently associate with GO terms of core T-cell exhaustion, and reveal potential driver genes for signatures.

A-B) Gene Ontology (GO) terms were surveyed among patients experiencing TIM-3 increase (top panels) and decrease (bottom panels): The top 20 GO biological process terms are plotted based on significance by

rank-based statistics **(A)** and normalized enrichment score by Gene Set Enrichment Analysis (GSEA) **(B)**. Obtained GO terms and the reference core T cell exhaustion GO terms[19] were summarized by REViGO to survey for associated terms:[26] Red color indicates relation to upregulated terms in core T cell exhaustion, while green indicates relation to reduced T cell exhaustion. The former is summarized by inflammatory response, cell chemotaxis and activation, cytokine biosynthesis and response to other organism, while the latter is summarized by cellular amino acid catabolism, lipid and steroid metabolism, acute inflammatory response and protein activation cascade (including T-cell receptor signaling). **C)** Leading Edge analysis of GSEA method was performed on three T-cell exhaustion/dysfunction signatures (middle, green separating lines),[19, 27, 29] each of which showed significant enrichment in TIM-3 increase group (not shown); and on the top 20 GO biological process terms among TIM-3 decrease (top left) and TIM-3 increase patients (bottom right), in order to survey potential drivers of gene signatures. Each category presents the top 25 leading edge genes differentially expressed in multiple signatures.

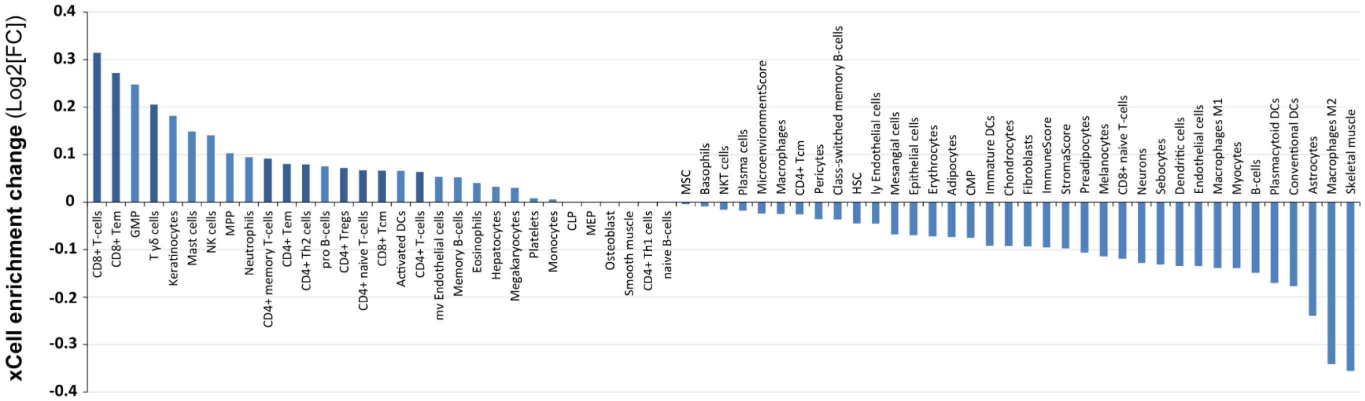

Fig. S4 Liikanen et al.

Supplementary Figure S4: Global transcriptome-based cell-type enrichment analysis indicates induction of T-cell immunity and mobilization of progenitors upon oncolytic adenovirus therapy.

Transcriptome-based cell-type enrichment analysis by xCell method was implemented on normalized batch-corrected gene expression data[28]: the global enrichment/reduction of cell-types is presented by mean log2-transformed fold change. Several stromal cell types, myeloid cells and epithelial (tumor) cells were reduced, whereas progenitor cells and especially T-cell subsets were induced (highlighted with darker color) following oncolytic adenovirus therapy. GMP, granulocyte-monocyte precursor; NK, natural killer; MPP, multipotent progenitor; Tem, effector-memory T cell; Treg, regulatory T cell; Tcm, central memory T cells; mv, microvascular; CLP, common lymphoid progenitor; MEP, megakaryocyte-erythroid progenitor; MSC, mesenchymal stem cell; NKT, natural killer T cell; HSC, hematopoietic stem cell; ly, lymphatic; CMP, common myeloid progenitor; DC, dendritic cell; TAM, tumor-associated macrophage.

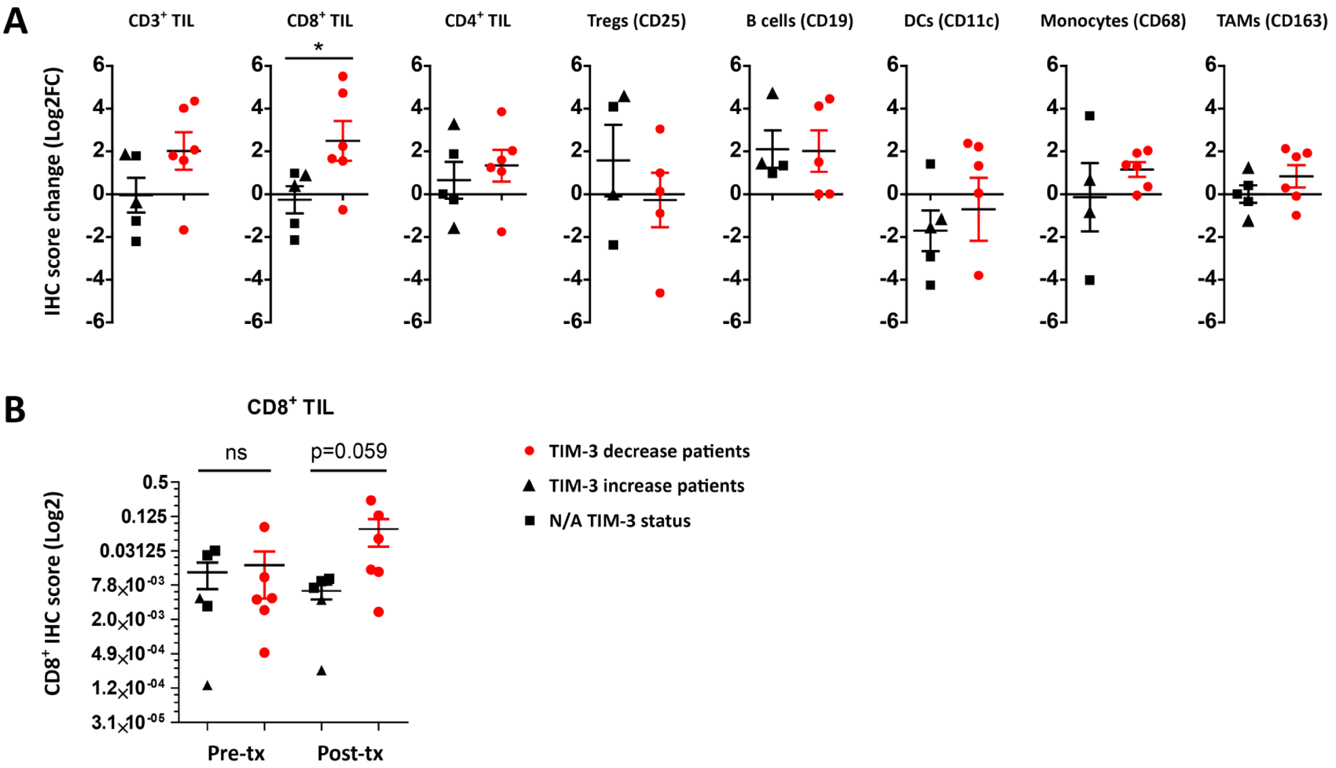

Fig. S5 Liikanen et al.

Supplementary Figure S5: Patients experiencing TIM-3 decrease in tumors show higher fold increase of CD8+ TIL but equal modulation of other immune cells following oncolytic adenovirus therapy.

Immunohistochemistry (IHC) analysis for indicated lymphocyte and myeloid cell markers were performed on available pre- and post-treatment tumor biopsy samples in 6 patients with TIM-3 decrease (red circle symbols) and 5 control patients (TIM-3 increase, triangle symbols; TIM-3 data not available, square symbols), presented as log2-transformed fold change. **A)** CD8+ TIL significantly induced in TIM-3 decrease patients over control patients (P = 0.044) following oncolytic adenovirus therapy, while other lymphoid or myeloid cell populations failed to show differences between groups. **B)** To assess absolute levels of CD8+ TIL infiltration per timepoint, the log2-transformed CD8a IHC scores were compared at pre- and post-treatment timepoints separately: Pre-treatment scores were equal between groups, whereas post-treatment scores trended for induction in TIM-3 decrease over control patients (P = 0.059). FC, fold change; Treg, regulatory T cell; DC, dendritic cell; TAM, tumor-associated macrophage; *, P<0.05; ns, not significant. Unpaired t-test in each.

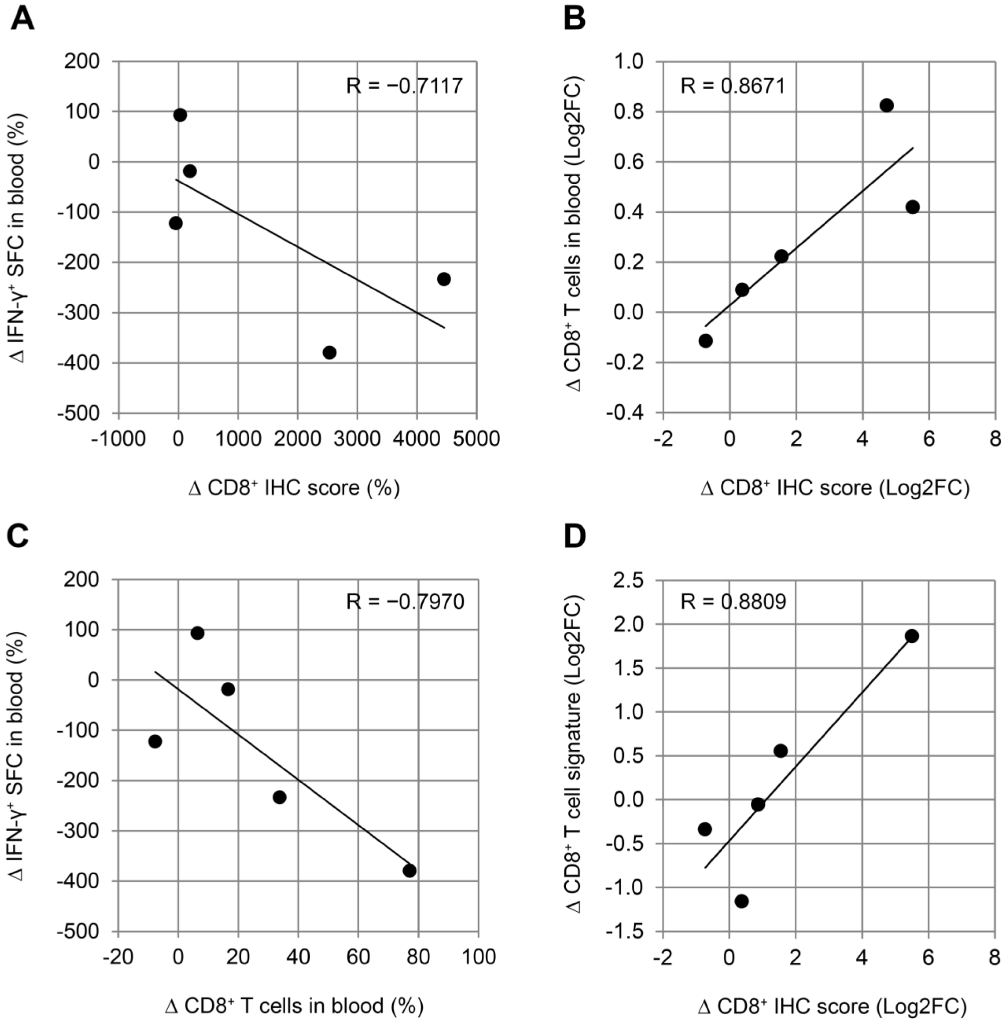

Fig. S6 Liikanen et al.

Supplementary Figure S6: **Immunological correlates between peripheral blood and tumor-site CD8$^+$ T cell data are compatible with trafficking phenomenon.**

Patients with evaluable peripheral blood samples and tumor biopsies were analyzed for circulating CD8$^+$ T-cell frequency (of parent CD3$^+$ immune cell population) and circulating IFN-γ producing cells (IFN-γ spot forming colonies, SFC, per million PBMCs) in conjunction with CD8a immunohistochemistry before and after oncolytic adenovirus treatment. **A)** Correlation of circulating IFN-γ producing T-cell change to CD8a immunohistochemistry score change, presented in percentages ($R = -0.7117$, $P = 0.1776$). **B)** Correlation of circulating CD8$^+$ T cell population change to CD8a immunohistochemistry score change, presented in log2-

transformed fold change (R = 0.8671, P = 0.057). **C)** Correlation of circulating IFN-γ producing T-cell change to total circulating $CD8^+$ T-cell population change in percentages (R = −0.7970, P = 0.1064). **D)** To verify association between immune cell proportion change in microarray *in silico* analysis and biological data, average tumor-site $CD8^+$ T-cell change by transcriptome-based methods[27-29] was correlated to CD8a immunohistochemistry score change, both presented in log2-transformed fold change (R = 0.8809, P = 0.0484; Pearson's R correlation coefficient, as displayed in all graphs).

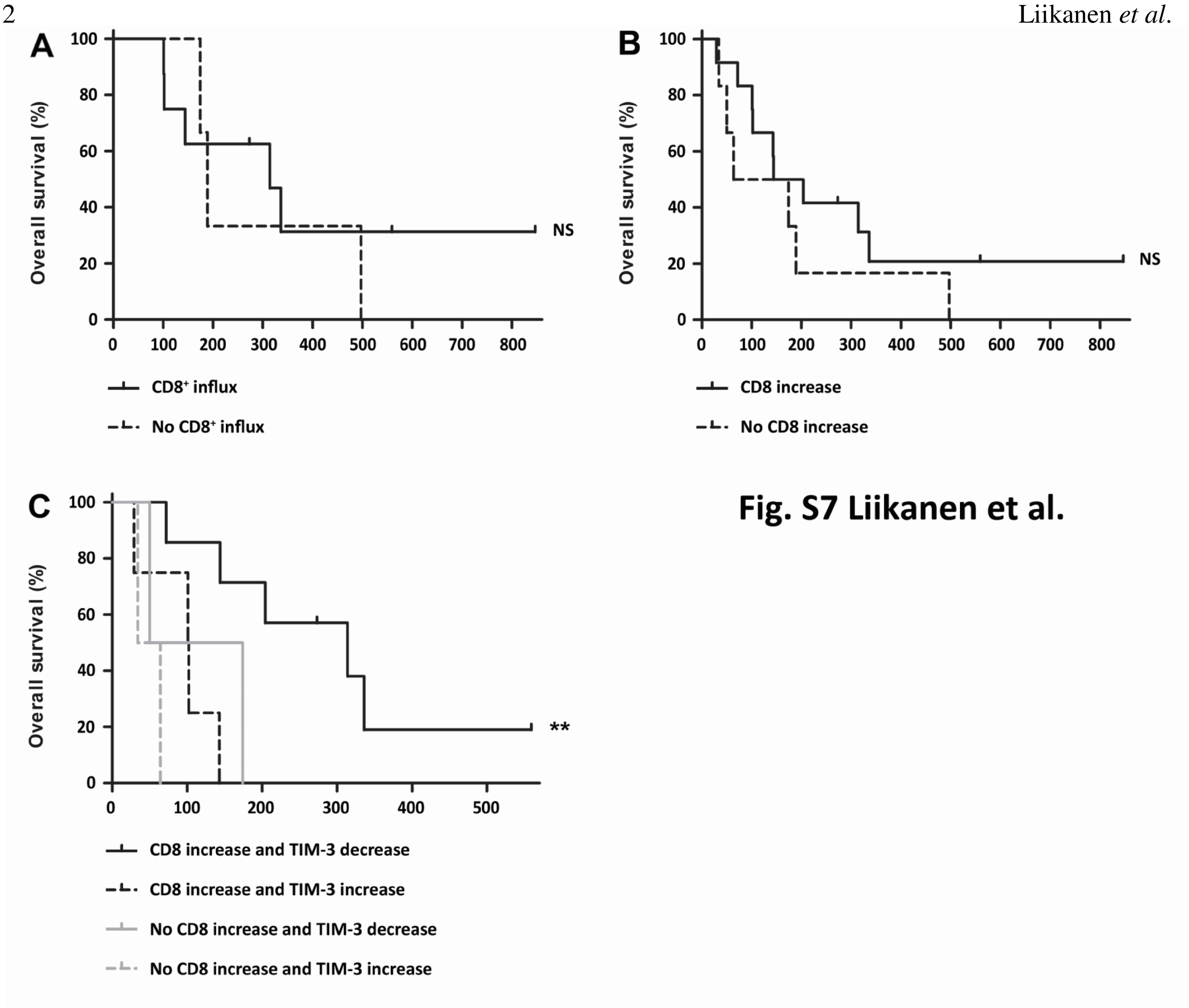

Supplementary Figure S7: Survival benefit after oncolytic immunotherapy is linked to combination of tumor-site CD8$^+$ T cell increase and concomitant TIM-3 decrease.

Patients were subgrouped for survival analysis either by CD8$^+$ TIL increase in immunohistochemistry **(A; and see Figure 5D)** or also by tumor-site *CD8A* expression increase **(B-C)**, with/without TIM-3 change status. CD8$^+$ T cell induction alone by either method failed to discriminate long-surviving patients **(A and B)**, whereas combination of TIM-3 decrease together with CD8$^+$ infiltration separated patients with the longest overall survival **(C; and see Figure 5D)**: Median OS of 336 days (95%-CI 177-495 days) as compared to 72 days (95%-CI 15-129 days) in patients without both of these phenomena by CD8$^+$ influx in figure 5D (P = 0.00235 *vs*. all, Log-Rank test), and median OS of 314 days (95%-CI 88-540 days) as compared to 64 days (95%-CI 0-135 days) in patients without both of these phenomena by both CD8$^+$ influx and *CD8A* expression increase in C (P = 0.00241 *vs*. all, Log-Rank test).

Supplementary Tables

Table S1. Individual patient characteristics, oncolytic virus treatments and response variables.

Patient ID	Age	Sex	ECOG score	Diagnosis	Sample material*	Oncolytic virus†	Transgene‡	Imaging response§	OS days	OS status (1=dead)	Tumor marker¶	Marker response	TIM-3 change
**C335**	**71**	**F**	**2**	**Colorectal cancer**	**P**								
**C335**	**71**	**F**	**2**	**Colorectal cancer**	**P**	**CGTG-401**	**CD40L**	**N/A**	**34**	**1**	**CEA**	**PD**	**INCR**
**C341**	**66**	**F**	**1**	**Colorectal cancer**	**B**								
**C341**	**66**	**F**	**1**	**Colorectal cancer**	**B**	**CGTG-401**	**CD40L**	**SD**	**314**	**1**	**CEA**	**SD**	**DECR**
**H333**	**61**	**F**	**2**	**Pancreatic cancer**	**A**								
**H333**	**61**	**F**	**2**	**Pancreatic cancer**	**A**	**CGTG-602**	**GMCSF**	**N/A**	**50**	**1**	**CA19-9**	**PD**	**DECR**
**H339**	**65**	**M**	**1**	**Pancreatic cancer**	**A**								
**H339**	**65**	**M**	**1**	**Pancreatic cancer**	**A**	**CGTG-602**	**GMCSF**	**N/A**	**29**	**1**	**CA19-9**	**PD**	**INCR**
**I398**	**25**	**F**	**1**	**Melanoma**	**B**								
**I398**	**25**	**F**	**1**	**Melanoma**	**B**	**CGTG-602**	**GMCSF**	**PD**	**174**	**1**	**N/A**	**N/A**	**DECR**
**K326**	**65**	**M**	**2**	**Lung cancer**	**P**								
**K326**	**65**	**M**	**2**	**Lung cancer**	**P**	**CGTG-201**	**N/A**	**N/A**	**72**	**1**	**N/A**	**N/A**	**DECR**
**M329**	**67**	**M**	**3**	**Mesothelioma**	**A**								
**M329**	**67**	**M**	**3**	**Mesothelioma**	**A**	**CGTG-401**	**CD40L**	**N/A**	**64**	**1**	**N/A**	**N/A**	**INCR**
**O38**	**36**	**F**	**1**	**Ovarian cancer**	**B**								
**O38**	**36**	**F**	**1**	**Ovarian cancer**	**B**	**CGTG-102**	**GMCSF**	**SD**	**336**	**1**	**CA125**	**SD**	**DECR**
**O279**	**62**	**F**	**2**	**Ovarian cancer**	**B**								
**O279**	**62**	**F**	**2**	**Ovarian cancer**	**B**	**CGTG-102**	**GMCSF**	**SD**	**101**	**1**	**CA125**	**PD**	**INCR**
**O340**	**75**	**F**	**0**	**Ovarian cancer**	**B**								
**O340**	**75**	**F**	**0**	**Ovarian cancer**	**B**	**CGTG-602**	**GMCSF**	**MR**	**559**	**0**	**CA125**	**CR**	**DECR**
**O391**	**53**	**F**	**2**	**Ovarian cancer**	**B**								
**O391**	**53**	**F**	**2**	**Ovarian cancer**	**B**	**CGTG-602**	**GMCSF**	**MR**	**143**	**1**	**CA125**	**PR**	**INCR**
**P407**	**71**	**M**	**2**	**Prostate cancer**	**B**								
**P407**	**71**	**M**	**2**	**Prostate cancer**	**B**	**CGTG-103**	**GMCSF**	**PD**	**273**	**0**	**PSA**	**MR**	**DECR**
**R356**	**40**	**F**	**1**	**Breast cancer**	**B**								
**R356**	**40**	**F**	**1**	**Breast cancer**	**B**	**CGTG-602**	**GMCSF**	**PD**	**102**	**1**	**CA15-3**	**PR**	**INCR**
**R367**	**59**	**F**	**1**	**Breast cancer**	**B**								
**R367**	**59**	**F**	**1**	**Breast cancer**	**B**	**CGTG-201**	**N/A**	**MR**	**204**	**1**	**CA15-3**	**MR**	**DECR**
**X373**	**57**	**F**	**2**	**Cervical cancer**	**B**								
**X373**	**57**	**F**	**2**	**Cervical cancer**	**B**	**CGTG-602**	**GMCSF**	**SD**	**144**	**1**	**CA125**	**PD**	**DECR**
R249#	61	F	1	Breast cancer	B								
R249#	61	F	1	Breast cancer	B	CGTG-102	GMCSF	N/A	497	1	N/A	N/A	N/A
R255#	77	F	1	Breast cancer	B								
R255#	77	F	1	Breast cancer	B	CGTG-102	GMCSF	N/A	847	0	N/A	N/A	N/A
R256#	50	F	1	Breast cancer	B								
R256#	51	F	1	Breast cancer	B	CGTG-102	GMCSF	N/A	189	1	N/A	N/A	N/A

* B = biopsied solid tumor, A = liquid biopsy of tumor-associated ascites, P = liquid biopsy of tumor-associated pleural effusion.

† After the baseline examination and biopsies, patients received the indicated oncolytic adenovirus, predominantly administered intratumorally into biopsied lesions, and/or in cases of liquid biopsies intraperitoneally or intrapleurally.[13] References for the oncolytic adenoviruses are specified in Materials and Methods.

‡ Immunostimulatory transgene expressed by the oncolytic adenovirus: GMCSF, granulocyte-macrophage colony stimulating factor; CD40L, CD40-ligand. N/A, not available (no transgene).

§ Modified PERCIST criteria was applied for metabolic response assessment by [(18)F]-fluorodeoxyglucose PET-CT (change in the maximum standardized uptake value [SUVmax] sum values): CR, complete response, disappearance of all tumors; PR, partial response, 30% or more reduction in the SUVmax sum values; MR, minor response, 10-29% reduction in the SUVmax sum values; SD, stable disease, metabolic tumor measurements not satisfying the criteria for

response or progression; PD, progressive disease, detection of new metastatic lesions (excluding lymph nodes potentially associated with active immunotherapy response) or increase in the SUVmax sum values by 20% or more, as previously reported.[33]

¶ Blood tumor markers, if elevated at baseline, were scored using the same percentage cutoffs as above: CEA, Carcinoembryonic antigen; CA19-9, Carbohydrate antigen 19-9; CA125, Cancer antigen 125; PSA, Prostate-specific antigen; CA15-3, Cancer antigen 15-3.

# Extension cohort for immunohistochemistry-based analyses; response data or TIM-3 status not available.

Table S2. Biological variables in TIM-3 expression change groups.

		**TIM-3 decr.** (n = 9)	**TIM-3 incr.** (n = 6)	**Total** (n = 15)	**Significance*** (TIM3 groups)	**Correlation*** (to ΔTIM-3)	
**Response variable**		*Average change post-treatment (± SEM)*			*Q-value*	*Pearson's R*	
TIM-3 change in tumor site	(FC)	−2.05x (±0.31)	+1.98x (±0.62)	−1.19x (±0.28)	***** (<0.0001)**	N/A	
Galectin-9 change in tumor site	(FC)	−1.35x (±0.14)	+1.19x (±0.32)	−1.12x (±0.17)	*** (0.034)**	**+0.657**	**
HMGB1 change in serum†	(mg/ml)	−0.05 (±0.32)	+1.21 (±0.28)	+0.52 (±0.28)	*** (0.017)**	**+0.626**	*
CAECAM1 change in tumor site	(FC)	−1.24x (±0.06)	−1.85x (±0.36)	−1.46x (±0.15)	ns. (0.179)	−0.239	ns.
**Baseline variable**		*Average (± SEM)*			*P-value*	*Pearson's R*	
TIM-3 baseline in tumor site	(expr.)	1924 (±367)	1731 (±605)	1846 (±315)	ns. (0.776)	−0.363	ns.
Galectin-9 baseline in tumor site	(expr.)	272.2 (±44.6)	231.1 (±28.3)	247.6 (±93.9)	ns. (0.427)	−0.092	ns.
HMGB1 baseline in serum†	(mg/ml)	1.124 (±0.39)	1.429 (±0.21)	1.263 (±0.23)	ns. (0.535)	+0.446	ns.
CAECAM1 baselin in tumor site	(expr.)	513.5 (±199)	326.9 (±70.7)	438.9 (±122)	ns. (0.475)	+0.021	ns.

* Tumor site microarray data were analyzed as described in Materials and Methods. Post *versus* pre-treatment expression change was examined for TIM-3 (*HAVCR2* gene) and its putative ligands: Galectin-9 expression (*LSGAL9* gene), HMGB1 serum levels, and CAECAM1 expression (*CAECAM1* gene); Patients were stratified into TIM-3 decrease (decr.) *vs*. TIM-3 increase (incr.) groups based on direction of TIM-3 expression change. Average fold change (FC), presented as fold decrease (< −1x) or fold increase (≥ +1x), were compared between TIM-3 groups (Q-value: FDR-corrected p-value), while each variable was also tested for linear correlation to TIM-3 change (ΔTIM-3; log2-transformed fold change) in the overall patient population. Pearson's correlation coefficient (R) was calculated by GraphPad Prism software.
† HMGB1 in serum was analyzed by ELISA for 6 and 5 patients with available baseline and post-treatment samples in TIM-3 decrease and increase groups, respectively. Two-tailed t-test (*P*-value) was used for statistical analysis of HMGB1 serum levels between TIM-3 groups.
FC, fold change; expr., baseline expression values; SEM, standard error mean; ns., not significant; *, $p < 0.05$; **, $p < 0.01$; ***, $p < 0.001$. Total N = 11 for HMGB1 serum data, N = 15 for others.

Supplementary Materials and Methods

Supplemental material BMJ Publishing Group Limited (BMJ) disclaims all liability and responsibility arising from any reliance placed on this supplemental material which has been supplied by the author(s) J Immunother Cancer

RNA microarrays

Total RNA was extracted from the samples using TRIzol Reagent (Life Technologies), purified with the RNeasy Mini kit (Qiagen, Hilden, Germany) and eluated into 30 µl of RNase-free water (Thermo Fisher Scientific, Waltham, MA). Quantity of the extracted RNA was evaluated spectrophotometrically using Nanodrop (Thermo Fisher Scientific) and quality was controlled by the Agilent 2100 Bioanalyzer (Agilent Technologies, Santa Clara, CA); Samples with RNA integrity score >8 were included in the analysis. For QC passed samples, genome-wide gene expression profiling was performed by labeling and hybridizing the RNA to the Illumina HumanHT-12 v4 Expression BeadChips arrays (Illumina, San Diego, CA), using the TotalPrep RNA Labeling Kit (Illumina) according to manufacturer's instructions; A total of 750 ng of purified biotinylated cRNA was hybridized for 18 hours at 58°C on a rocker. Finally, BeadChips were washed, blocked, stained with streptavidin-Cy3 and scanned with Illumina iScan (Illumina) according to manufacturer's protocols. Genome Studio software (Illumina) was used to control the quality of the data.

Microarray data analysis

Raw probe intensity values were first summarized and exported with Illumina probe annotations using Illumina BeadStudio. Then, the non-normalized, non-background corrected data were quantile-normalized and log2 transformed, followed by removal of chip-dependent batch effects using the ComBat method . Data processing and analysis was performed using Bioconductor in R. Probes were annotated according to information from Ensembl; Probes mapped to the same gene were averaged into a single expression estimate, while probes pointing to multiple genes or left without a gene information were removed. For statistical testing, post- *versus* pre-treatment data were first compared per patient to account for interpatient variation such as sample material (matched sample material of each biopsy pair) as well as for other biological and clinical baseline variation such as tumor type. Finally, the normalized differential expression data was divided into analytical groups according to direction of TIM-3 expression change and analyzed by rank-based statistics, implemented in RankProd Bioconductor package, to assess global gene expression change differences between TIM-3 groups. Results were further corrected for multiple comparisons by the end analyses described below.

Gene Ontology (GO) term analysis (MbdSig database, v.2015) was separately performed among TIM-3 upregulation and TIM-3 downregulation groups using the permutation test by EnrichR package in R. Additionally, enrichment of the GO terms, together with the reported core transcriptional $CD8^+$ T cell exhaustion signature obtained from Bengsch *et al*.[19] were surveyed on the pre-ranked differentially expressed genes (ranked based on log2 fold change) between TIM-3 groups using the Gene Set Enrichment Analysis (GSEA v.4.0.3, Broad Institute, MIT): In all analyses 1000 permutations were used to determine normalized enrichment scores and FDR-corrected Q-values for gene signatures. After the GO enrichment analyses, generated results were ranked based either on significance (EnrichR) or normalized enrichment score (GSEA), and were summarized by REViGO to survey for terms associated either with upregulated T cell exhaustion signature or downregulated geneset in exhaustion, as reported.[19, 26] In addition, both the upregulated and downregulated gene signatures of the core T cell exhaustion were also assessed for significant accumulation between TIM-3 groups by Chi^2 tests (volcano plots).

For transcriptome-based cell-type analyses, three methods were implemented on patient gene expression data: 1) xCell method[28] 2) ImmuCellAI method[29] 3) Tumor Immune Dysfunction and Exclusion (TIDE) method.[27] For the xCell and ImmuCellAI methods, normalized batch-corrected expression data was submitted and the raw cell-type estimates were analysed by mean log2-transformed fold change using the recommended signal threshold of $P<0.2$ (xCell) or by cell-type abundance score change (post – pre; ImmuCellAI). For the TIDE method, the batch-corrected pre-treatment biopsy data were used as baseline background signal and subtracted from each matched-biopsy post-treatment sample (post – pre oncolytic virus therapy onset) and the raw output scores were presented. Thus, the experimental setup addresses pre-conditioning of tumors by oncolytc adenovirus for potential checkpoint blockade therapy at 8 weeks median. The TIDE algorithm was conducted selecting other cancer type, and comparable results were obtained whether previous immunotherapy or no previous immunotherapy was selected for the response prediction rule. Of note, all patients were immunotherapy-naïve before the onset of oncolytic adenovirus therapy.

RT-qPCR

Total RNA was extracted from mouse tumors using QIAGEN RNeasy Plus Kit. First-strand cDNA was synthesized using the iScript cDNA Synthesis kit (cat.#1708890; Bio-Rad). Real-time qPCR was carried out using synthesized cDNA, HOT FIREPol EvaGreen qPCR Mix (cat.#08-250000S; Solis Biodyne), SensiMixSYBR Master Mix (Origene, cat.#QP100002), Havcr2_Fwd- ACAGACACTGGTGACCCTCCAT and Havcr2_Rev- CAGCAGAGACTCCCACTCCAAT (Origene, cat.#MP206036)

Flow cytometry

Preclinical tumor samples were stained according to manufacturer's instructions with the following commercial antibodies validated by the supplier (all from eBioscience unless otherwise noted): CD3e-PE (cat.#12-0031-82), CD45-PerCP-Cy5.5 (cat.#45-0451-82), CD8b-FITC (cat.#11-0083-85), CD4-PeCy7 (cat.#25-0041-82), TIM-3-APC (cat.#17-3109-42), NK1.1-FITC (cat.#11-5941-81), CD11b-PerCP-Cy5.5 (BD, cat.# 550993), CD11c-FITC (BD, cat.#553801), CD3e-FITC (cat.#11-0031-82), CD8a-APC (cat.#17-0081-82), CD4-PE-Cy7 (cat.#25-0041-82), CD44-BV605 (BD, cat.#563058), CD45-V500 (BD, cat.#560777), TIM-3-PE (cat.#12-5870-82), LAG3-PerCP-Cy5.5 (BD, cat.#564673), PD-1-BV421 (BD, cat.#748268). Single-cell suspensions were stained with monoclonal antibodies for 30 minutes at +4°C. The labeled cells were centrifuged at 500 rcf for 5 min, washed twice, and the pellet was resuspended in Flow Cytometry Staining Buffer (eBioscience). Stained samples were then analyzed on BD Accuri C6 or LSRFortessa X-20 flow cytometer (both from BD Biosciences). FlowJo software v.10.5.3 (Tree Star, Ashland, OR) was used to analyze preclinical tumor samples. Fluorescence minus one control was included for activation-related markers and gating was performed accordingly.

For clinical peripheral blood samples, the peripheral blood mononuclear cells (PBMCs) were first extracted from collected whole blood samples by gradient centrifugation and then stored in CTL-Cryo medium (Cellular Technology, Shaker Heights, OH) at −140 °C. For surface marker analysis, cells were washed by transferring frozen samples to 10 ml of phosphate-buffered saline. Samples were then resuspended in 2 ml of RPMI-1640, 10% FBS, 1% L-Glutamine, 1% Pen/Strep solution and incubated overnight at 37°C. After incubation, samples were washed and resuspended in stain buffer (phosphate-buffered saline, 2% FBS), and stained with conjugated antibodies against cell surface markers CD3-Pe-Cy7, CD4-PerCP-Cy5.5, CD8a-

APC-H7, and CD25-APC (all from BD Biosciences). Cells were then washed with CellWash buffer, fixed using Cytofix/Cytoperm solution and stained for intracellular marker FoxP3-V450 (all from BD Biosciences) to examine regulatory T cells (no differences, not shown). After staining, cells were washed with CellWash buffer and suspended in CellfIX buffer (BD Biosciences). Flow cytometry analysis was performed using LSR Fortessa X-20 and the data were analyzed with FACSDiva v.6.0 Software (BD Biosciences).
